# Development of an Innovative Nanosystem Based on Functionalized Albumin and Oxidized Gellan for the Synergistic Delivery of Curcumin and Temozolomide in the Treatment of Brain Cancer

**DOI:** 10.3390/gels11090708

**Published:** 2025-09-03

**Authors:** Camelia Elena Iurciuc (Tincu), Gabriela Vochița, Daniela Gherghel, Cosmin-Teodor Mihai, Silvia Vasiliu, Ștefania Racoviță, Anca Niculina Cadinoiu, Corina Lenuța Logigan, Mihaela Hamcerencu, Florin Mitu, Marcel Popa, Lăcrămioara Ochiuz

**Affiliations:** 1 Department of Natural and Synthetic Polymers, “Cristofor Simionescu” Faculty of Chemical Engineering and Protection of the Environment, “Gheorghe Asachi” Technical University, 73, Prof. Dr. Docent Dimitrie Mangeron Street, 700050 Iasi, Romania; camelia_tincu83@yahoo.com (C.E.I.); savincorina@yahoo.com (C.L.L.); 2 Department of Pharmaceutical Technology, Faculty of Pharmacy, “Grigore T. Popa” University of Medicine and Pharmacy, 16, University Street, 700115 Iasi, Romania; ochiuzd@yahoo.com; 3 Institute of Biological Research Iasi, Branch of National Institute of Research and Development for Biological Science, 47, Lascar Catargi Street, 700107 Iasi, Romania; gabriela.vochita@icbiasi.ro (G.V.); daniela_gherghel@yahoo.com (D.G.); 4 Department of Medical Genetics, Faculty of Medicine, “Grigore T. Popa” University of Medicine and Pharmacy, 16, University Street, 700115 Iasi, Romania; cosmin-teodor.mihai@umfiasi.ro; 5 Praxis Medical Investigations, Moara de Vant Street, No. 35, 700376 Iasi, Romania; 6 “Petru Poni” Institute of Macromolecular Chemistry, Grigore Ghica Voda Alley, No. 41A, 700487 Iasi, Romania; vasisilv@yahoo.com (S.V.); stefania.racovita@icmpp.ro (Ș.R.); 7 Faculty of Dental Medicine, “Apollonia” University of Iasi, 11, Pacurari Street, 700511 Iasi, Romania; jancaniculina@yahoo.com; 8 Laboratoire de Photochimie et Ingénierie Macromoléculaires—Ecole Nationale Supérieure de Chimie de Mulhouse, Université de Haute Alsace, 3 Rue Alfred Werner, 68093 Mulhouse Cedex, France; mhamcerencu@yahoo.com; 9 Department of Medical Specialties (I), Faculty of Medicine, “Grigore T. Popa” University of Medicine and Pharmacy, University Street, 700115 Iasi, Romania; mitu.florin@umfiasi.ro; 10 Academy of Medical Sciences, I. C. Brătianu Street, Nr. 1, Sector 3, 030167 Bucharest, Romania; 11 Academy of Romanian Scientists, Ilfov Str., Nr. 3, Sector 5, 050045 Bucharest, Romania

**Keywords:** functionalization of human serum albumin, HSA-based nanoparticles, overcoming blood–brain barrier, oxidized gellan crosslinking, target delivery, synergic effect of co-encapsulated curcumin and temozolomide

## Abstract

Treating brain cancer remains challenging due to the blood–brain barrier (BBB) and the systemic toxicity of chemotherapy. This study focuses on developing human serum albumin (HSA) nanoparticles modified with low-molecular-weight protamine (LMWP) to improve crossing the BBB and enable targeted delivery of curcumin and temozolomide (TMZ). Nanoparticle stability was enhanced by crosslinking with aldehyde groups from oxidized gellan (OG). The successful attachment of LMWP to HSA at the thiol group of Cys34 was confirmed through FT-IR and ^1^H-NMR analyses. Most self-assembled nanoparticles were smaller than 200 nm in diameter. Curcumin showed higher encapsulation efficiency than TMZ. In vitro drug release was pH-dependent: curcumin released more at pH 7.4, while TMZ release was better at pH 4. Higher crosslinking degrees reduced drug release. Cytotoxicity assays on V79-4 (normal) and C6 (glioma) cell lines showed increased apoptosis and significantly lower IC_50_ values for co-encapsulated formulations, indicating a synergistic effect. Curcumin’s antioxidant activity was maintained and protected from UV degradation by the polymer matrix. The parallel artificial membrane permeability assay (PAMPA) confirmed that the functionalized formulations with co-encapsulated drugs could cross the BBB. Hemocompatibility studies indicated a favorable profile for intravenous use.

## 1. Introduction

Glioblastoma (GBM) is the most common type of primary malignant brain tumor, representing 16% of all primary brain and central nervous system cancers [1]. Its treatment requires a multidisciplinary approach. The standard treatment now involves maximal safe surgical removal, followed by radiotherapy and simultaneous administration of temozolomide (TMZ), an oral chemotherapy drug, along with subsequent adjuvant chemotherapy using TMZ [2]. Due to its high invasiveness, radical resection of the primary tumor mass is not always curative, as infiltrative tumor cells often remain in the surrounding brain, causing disease progression or recurrence [3,4,5].

Temozolomide (TMZ) is the standard chemotherapy for treating GBM, either alone or in combination with radiotherapy [6]. However, TMZ has the disadvantage of damaging hematopoietic stem cells, leading to dose-dependent hematological toxicity, which results from its non-selective attack on DNA. Additionally, TMZ has limited solubility in physiological conditions and is rapidly hydrolyzed, reducing its effectiveness as an antitumor drug (with a half-life of 1.8 h), which requires frequent dosing [7,8,9]. Prolonged use of TMZ can lead to drug resistance and a poor response to future treatments, causing tumor recurrence in 60–75% of cases [9,10,11,12].

Co-administration of two anticancer agents with different mechanisms of action can produce a synergistic effect, resulting in a treatment that is significantly more effective than using a single drug alone [13]. Recent clinical trials have demonstrated that curcumin may be effective in both preventing and treating various types of cancer. It also affects critical signaling pathways involved in cell survival, carcinogenesis, and apoptosis [14,15]. When combined with chemotherapy drugs such as carboplatin, etoposide, vincristine, doxorubicin, cisplatin, and TMZ in cancer cell cultures, curcumin has been shown to reduce cell viability, modify the cell cycle, and increase the rate of cell apoptosis compared to using these drugs alone [16,17]. Additionally, it has a protective effect against reactive oxygen species (ROS) [18,19].

Polyphenols provide several health benefits; however, their effectiveness can be limited due to poor absorption, rapid metabolism, and low water solubility. They undergo degradation through various enzymatic pathways, including glucuronidation, sulfation, and the cytochrome P450 system, leading to rapid metabolism and elimination from the body. Despite these challenges, polyphenols exhibit low toxicity, which allows the use of higher doses [20]. Efforts to address these disadvantages have included the development of formulations such as micelles, liposomes, nanoparticles, complexes, and emulsions. Polymer matrices can protect polyphenols from harmful environmental conditions, thereby extending the half-life of these bioactive compounds and improving their bioavailability both in vitro and in vivo [13,14,21].

Drug delivery systems for brain cancer therapy have been developed and should target the tumor, crossing the blood–brain barrier (BBB) [22,23], which requires lipophilic molecules with a molecular weight below 400 Da. Molecules that do not meet these conditions can only cross the BBB if transport systems or receptors facilitate their passage [24,25]. The primary mechanisms through which drugs overcome the BBB include absorption-mediated transcytosis, carrier-mediated transcytosis, and receptor-mediated endocytosis. The blood–brain tumor barrier (BBTB), like the BBB, is located between brain tumor tissues and microvessels formed by specialized endothelial cells, which limit the release of hydrophilic molecules into the tumor tissue. Proposed strategies for targeting the BBTB mainly focus on receptors expressed on tumors, such as epidermal growth factor receptors and integrins [16,25,26]. Hydrophobic or lipophilic nanoparticles are more likely to adhere to overexpressed proteins than those with hydrophilic surfaces [27].

Additionally, particle size is also essential, ranging from 3 to 200 nm [28]. Active recognition of target cells can be achieved by conjugating targeting ligands to the surface of nanoparticles [29]. However, less than 1% of nanoparticles functionalized with different ligands typically overcome the BBB [30,31].

Various delivery systems for TMZ, such as liposomes and solid lipid nanoparticles (NPs), have been developed and tested to ensure controlled and sustained release of the encapsulated drug, aiming to improve its therapeutic index [32,33,34,35]. The efficiency of these formulations has been limited because of inadequate targeting of TMZ to tumor cells.

This research aims to develop a delivery system that employs functionalized human serum albumin crosslinked with oxidized gellan (OG) through the self-assembly method. The delivery system will co-encapsulate hydrophobic drugs that exhibit a synergistic effect, allowing them to overcome the BBB and treat brain cancer effectively. A peptide will facilitate intracellular diffusion, and the self-assembled albumin nanoparticles will be modified using low-molecular-weight protamine (LMWP) to enhance the diffusion coefficient of the HSA-based nanoparticles into the brain.

Tumors, due to their rapid growth, require a significant amount of nutrients. We choose to obtain albumin-based nanoparticles because albumin serves as a vital nutrient source for the body. Typically, albumin is prevented from entering the brain, but in certain situations, such as the presence of a brain tumor, the absorption of albumin by tumor tissues increases [36]. It was demonstrated that the binding of nab-paclitaxel (albumin-bound paclitaxel) to the endothelium increased treatment efficiency by 9.9 times, and paclitaxel was transported 4.2 times more effectively compared to Cremophor EL-paclitaxel [37,38]. Nab-paclitaxel has demonstrated superior antitumor efficacy and improved tolerability compared to Taxol in a phase III clinical trial [39]. The albumin-based nanodelivery system received approval from the US Food and Drug Administration (FDA) for the treatment of breast cancer in 2005 [40], lung cancer in 2012 [41], and pancreatic cancer in 2013 [42,43]. The process by which albumin accumulates in tumors remains unclear [44]. Research indicates that albumin binds to the 60 kDa glycoprotein receptor (gp60, gene name albondin) located on vascular endothelial cells, facilitating its transport into the tumor interstitium through transcytosis [45,46]. Additionally, the secreted protein acidic and rich in cysteine (SPARC) captures albumin within the tumor stroma, which is associated with tumor-specific albumin uptake [47]. Albumin’s extended half-life is attributed to its protection against intracellular degradation by the FcRn receptor, which enables it to re-enter the bloodstream through lymphatic circulation, thereby extending its half-life [48,49,50].

Albumin can incorporate hydrophobic drugs, enhancing the efficiency of drug delivery to targeted areas. However, poor tissue diffusion remains a significant obstacle in delivering drugs to cancer cells. The diffusion coefficient for macromolecules through the brain tissue is estimated to be 10^−6^ cm^2^/s, which means it would take approximately three days for a substance to diffuse 1 mm. To address this challenge, a peptide known as low-molecular-weight protamine (LMWP), which can enhance the permeability of nanoparticles into cells, was used to functionalize these nanoparticles by covalently attaching the LMWP to the sulfhydryl groups of albumin [51]. A delivery system based on albumin nanoparticles modified with the peptide LMWP was designed to co-encapsulate paclitaxel (PTX) and fenretinide (4-HPR), thereby enhancing cellular permeability and overcoming the BBB. The self-assembly of denatured albumin enabled the co-encapsulation of these two hydrophobic drugs in an environmentally friendly way, eliminating the need for harmful crosslinking agents. The LMWP functionalization significantly improved intratumoral infiltration and enhanced the antitumor efficacy against U87 cells [36].

In order to improve the stability of LMWP-functionalized albumin nanoparticles formed through self-assembly, we performed a covalent crosslinking reaction between the amine groups of albumin and the aldehyde groups of oxidized gellan. Compared to other crosslinkers, oxidized gellan offers an ideal balance of efficiency and biocompatibility, enabling the controlled formation of Schiff bases between the aldehyde groups of gellan and the amine groups of albumin, resulting in a stable polymeric network that maintains the protein’s native structure and its ability to self-assemble. Overall, oxidized gellan provides adjustable gelling properties in the presence of divalent ions, ensures slow enzymatic degradation to prevent toxic accumulation, and eliminates the need for using traditional crosslinkers like glutaraldehyde, which poses significant cytotoxicity risks.

To conduct research on the development of albumin nanoparticles using OG, a systematic preliminary study was necessary to investigate the formation of these hydrogels, as detailed in [52]. The objective was to determine the conditions that enable crosslinking between albumin and OG. A previous research article discussed the prior oxidation of gellan using sodium periodate (NaIO_4_) and the physicochemical characterization of the resulting product [52].

The primary objective of this research is to develop a delivery system that can effectively overcome the BBB for treating brain tumor conditions. This system consists of nanoparticles based on albumin that are functionalized with low-molecular-weight protamine (LMWP), achieved through self-assembly, and loaded with two antitumor therapeutic agents: curcumin and TMZ.

The originality of this research lies in the development of albumin nanoparticles that are functionalized with LMWP crosslinked with OG using the self-assembly method, as well as in the co-encapsulation of TMZ and curcumin within the resulting nanoparticles.

The expected results of the study included confirming the structure of the HSA–LMWP conjugate through 1H-NMR, FT-IR, and UV-Vis spectroscopy; developing stable, biocompatible nanoparticles capable of co-encapsulating temozolomide and curcumin as hydrophobic drugs with synergistic effects; and demonstrating a controlled release profile under physiological conditions (pH 4 and pH 7.4). The study also aimed to establish a favorable biological profile characterized by low cytotoxicity, hemocompatibility, antioxidant activity, and the ability to induce apoptosis. Another anticipated result is an increase in permeability in the in vitro blood–brain barrier model (PAMPA-BBB test), highlighting the role of LMWP in enhancing nanoparticle permeability, enabling transport through the BBB, and targeting tumor tissue in the brain. Additionally, the stability of curcumin encapsulated in these nanoparticles was evaluated after exposure to various environmental conditions, including pH and light.

## 2. Results and Discussion

### 2.1. Obtaining Low-Molecular-Weight Protamine (LMWP) and Determining the Number of Moles of Free Amine Groups

An enzymatic approach utilizing thermolysin was employed to modify protamine for its proteolytic cleavage, resulting in a low-molecular-weight protamine [53]. Thermolysin cleaves protamine proteolytically at isolated peptide bonds adjacent to hydrophobic residues (Leu, Ile, Val, Phe), resulting in smaller peptide fragments. Each cleavage generates a new N-terminus with a free α-amine group [54,55].

Following the cleavage of protamine by thermolysin, albumin fractions were gathered, revealing that the quantity of free amine groups increases as molecular weight decreases. This increase takes place due to the formation of new amine groups at the chain fragment ends. Thus, with a reduction in the molecular weight of the fragments (indicating more cleavages per molecule), the overall number of free amine groups recorded per mole of substance also increases. LMWP is produced from the enzymatic breakdown of protamine using thermolysin. The literature suggests various drug delivery strategies that employ delivery systems functionalized with LMWP to overcome significant biological barriers, such as skin and intestinal mucosa [56]. These systems have also been analyzed for their potential to address intratumoral heterogeneity and transporter-mediated efflux mechanisms [57]. Previous research shows that using LMWP-modified drug–albumin conjugates can reduce the effects of efflux associated with drug resistance and improve therapeutic efficacy in anticancer treatments [58].

Samples of 1 mL were taken, and the protein concentration was determined using the Lowry method. After purification, the product was lyophilized. During the separation, 12 aliquots of 1 mL fractions of protamine were collected at different times, and the ninhydrin solution test was performed on several fractions of modified protamine (Table 1). Figure S1 in the Section 4 illustrates the procedure for protamine modification. Table 1 presents the results obtained from determining the number of moles of free amine groups in protamine and from several fractions collected during the purification of low-molecular-weight protamine. The total number of moles of free amine groups in HSA is 6.31 ± 0.7 × 10^−4^. Table 1 illustrates that the amount of free amine groups in the examined fractions of enzymatically modified protamine increases progressively from one fraction to the next, due to the production of LMWP.

### 2.2. Modification of Human Serum Albumin with Low-Molecular-Weight Protamine

**Figure 1:**
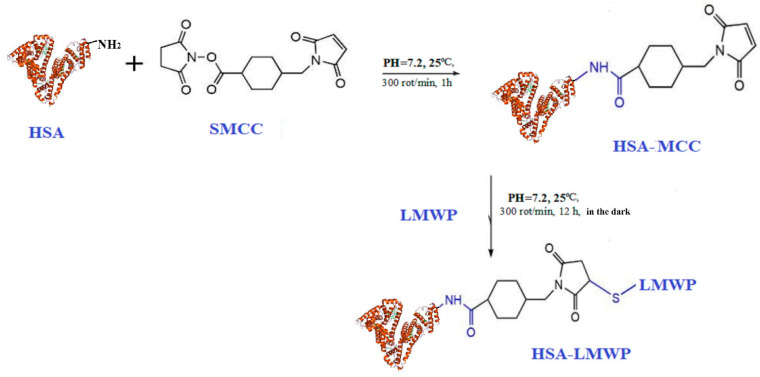
Schematic illustration of the albumin functionalization process with LMWP, using SMCC as the activating agent.

The modification of HSA with LMWP was performed to enhance the cellular permeability of the nanoparticles. The reactions involved in this functionalization process are illustrated in Figure 1, which provides a schematic overview of the chemical modifications that occur during this process.

UV-Vis absorption spectra confirm the formation of the HSA–LMWP conjugate. Figure S2 displays the UV-Vis absorption spectra recorded for HSA, HSA-SMCC (after purification), LMWP, and the final product HSA–LMWP. The UV-Vis spectrum of native HSA displays a characteristic band around 280 nm, which is attributed to the π→π transitions of aromatic residues, particularly phenylalanine, tyrosine, and tryptophan. After activation of the SH group with SMCC and purification, this peak shifts to 272 nm. In the final product, the HSA–LMWP conjugate, the peak appears at 270 nm.

Shifts towards shorter wavelengths are recognized as indicators of changes in the local environment around aromatic chromophores, which typically result from conformational changes or upon exposure to a polar solvent, such as DMSO, in this case. Similar spectral behaviors have been observed in situations involving interactions between HSA and small molecules such as 5-fluorouracil, where binding leads to an analogous shift and alterations in absorption intensity, supporting the notion of actual complex formation rather than just a simple combination of components [59].

In addition to the shift in the aromatic band, the HSA–LMWP conjugate displays a distinct peak around 220 nm, which is not present in native HSA. This peak is attributed to the absorption of amide bonds (n→π transitions) in the UV region of approximately 180–230 nm. This absorption peak is typical of peptides and is particularly observable in short, arginine-rich peptides like LMWP, which does not contain any aromatic residues [60]. The free LMWP has a maximum UV absorption peak around 230 nm and does not absorb at 280 nm. In the spectrum of the HSA–LMWP conjugate, the presence of an absorption band at 220 nm, along with the shift of the aromatic band, indicates that LMWP is covalently bound to HSA rather than existing as a free peptide.

#### 2.2.1. ^1^H-NMR Spectroscopy

**Figure 2:**
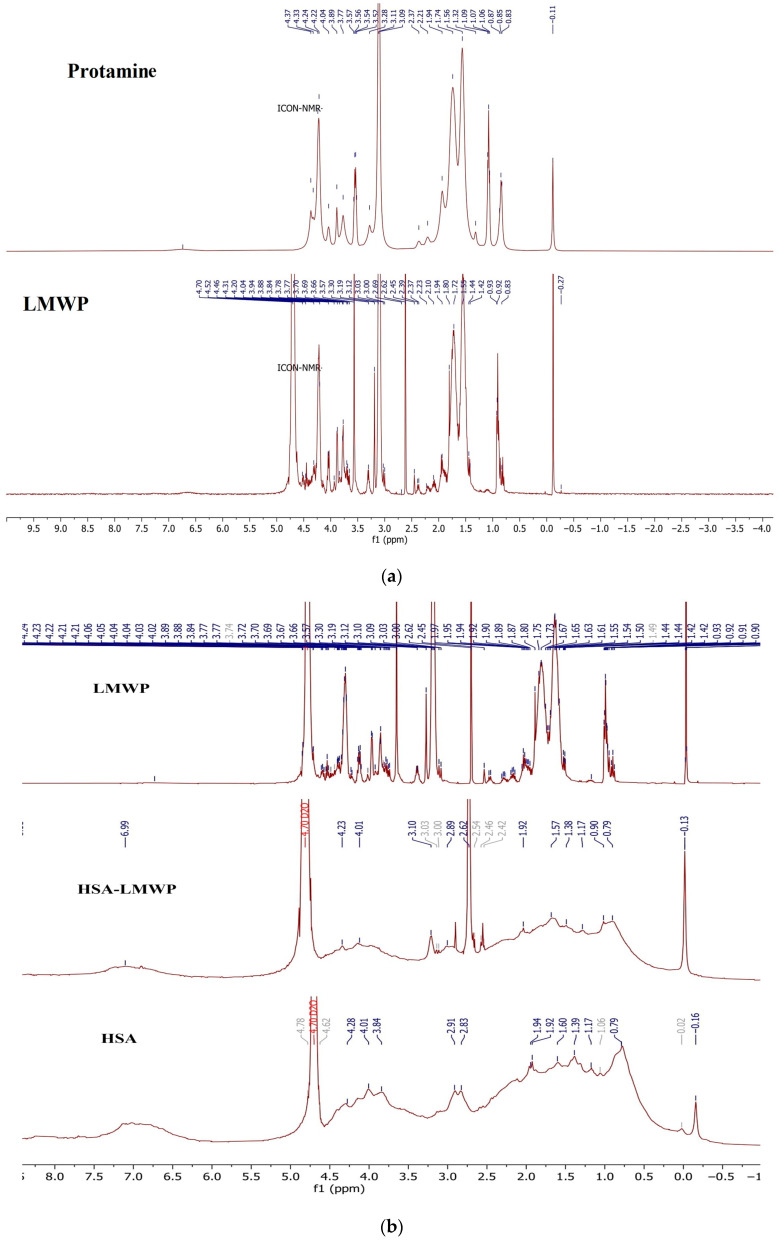
^1^H-NMR spectra obtained for standard protamine and LMWP (**a**), along with HSA, LMWP, and LMWP-modified albumin (**b**).

In order to confirm the functionalization of HSA with LMWP, ^1^H-NMR analysis was performed for standard protamine, LMWP, HSA, and LMWP-modified HSA. As illustrated in Figure 2, the spectra reveal characteristic chemical shifts that confirm the conjugation.

Modifying the single free sulfhydryl group at position Cys-34 of HSA offers an effective strategy for the specific conjugation of thiol-reactive ligands. In this study, we activated the thiol group (–SH) using the coupling agent SMCC (succinimidyl-4-(N-maleimidomethyl)cyclohexane-1-carboxylate) and then conjugated it with an LMWP peptide, which enhances intracellular diffusion. Salmon protamine sulfate consists of four primary peptides, each containing 30–32 residues of the amino acids arginine, serine, proline, isoleucine, alanine, glycine, and valine [61]. The structure of protamine sulfate and LMWP was examined using ^1^H NMR spectroscopy. The protamine sulfate spectrum (Figure 2a) displays the most significant signals from arginine at 1.32 ppm, 1.56 ppm, 3.09 ppm, and 4.22 ppm. Conversely, the LMWP spectrum shows characteristic signals for arginine at 1.55 ppm, 1.72 ppm, 2.65 ppm, 3.19 ppm, 3.6 ppm, and 4.2 ppm. The differences in signal intensity in the LMWP spectrum indicate that protamine modification has taken place in the presence of thermolysin.

^1^H NMR spectroscopy was employed to evaluate the structural changes that occur when LMWP conjugates with HSA, specifically focusing on identifying the binding site. The comparative analysis of spectra obtained from native HSA, HSA–LMWP, and free LMWP aimed to determine the presence, absence, or modification of signals related to the reactive thiol group of the Cys34 residue, which is the only free thiol group among the cysteine residues in human serum albumin. In the ^1^H-NMR spectrum of native HSA, the region between δ = 2.0 and 2.2 ppm—associated with the thiol group or the functional reactivity of the Cys34 residue—displays a low-intensity and poorly defined signal centered around a δ value of approximately 2.05 ppm. This signal corresponds to the mercaptalbumin form and aligns with findings in the literature. Observations indicate that protons near the free SH group create resonances within this range [62,63,64,65]. This signal is absent in the HSA–LMWP spectrum, suggesting a covalent modification of Cys34. The absence of a signal around δ = 2.05 ppm indicates that the thiol group has been modified due to a conjugation reaction; this effect has also been noted in earlier research on the interaction of cisplatin with the Cys34 residue [65].

The presence of a pronounced and distinctly defined signal at δ = 2.62 ppm in the HSA–LMWP spectrum, which is not present in the native HSA spectrum but is detectable in free LMWP, indicates that it is derived from the LMWP component of the HSA–LMWP conjugate. As shown in the literature, the signal at δ = 2.62 ppm is attributed to the methylene protons of arginine residues [66,67], indicating that this segment of the peptide is involved in the covalent binding of LMWP to albumin. Furthermore, the observation that the characteristic LMWP signal is transferred and remains unchanged in the spectrum of functionalized albumin suggests considerable local mobility of the attached moiety and supports the formation of a stable covalent bond, likely between the maleimide group of SMCC and the thiol of residue Cys34.

The ^1^H NMR spectra obtained demonstrate that LMWP binds specifically to the Cys34 residue of HSA. The disappearance of the free thiol signal (δ ~2.05 ppm), the emergence of a new signal at δ = 2.62 ppm—which is also found in free LMWP—and the preservation of the overall HSA spectrum structure support the concept of a localized alteration at the Cys34 site. These findings are consistent with previous research highlighting the enhanced reactivity of this group [64,65]. Therefore, ^1^H NMR spectroscopy effectively verifies the incorporation of LMWP into the protein structure and identifies the exact binding or conjugation site.

The aliphatic region of the ^1^H NMR spectrum for native HSA (δ 0.79–1.94 ppm) exhibits broad signals corresponding to the methyl and methylene protons from hydrophobic residues, such as Val and Leu, which are located at δ 0.87–1.00 ppm and δ 1.30–1.50 ppm, respectively [68]. In the free LMWP spectrum, the distinct peaks in the δ region 1.60–1.80 ppm are linked to the β/γ methylene protons of arginine [68]. In the HSA–LMWP spectrum, these signals overlap with the aliphatic signals of HSA, appearing broader and weaker, which indicates a decrease in the mobility of the protein-bound peptide [58]. The methyl chemical shifts, which changed by about 0.01 ppm after conjugation, indicate slight alterations in the spatial configuration and molecular interactions near these protons due to the bonding of LMWP with albumin.

In the aromatic region (δ7.0–8.5 ppm), the ^1^H NMR spectra of HSA and HSA–LMWP show clear signals for phenylalanine, tyrosine, and histidine (His at δ7.1–7.3 ppm and δ8.1–8.3 ppm), Phe at δ7.3 ppm, and Tyr in the range of δ6.9–7.1 ppm [68,69]. LMWP, which does not contain any aromatic residues, does not generate signals in the aromatic region. The aromatic profile of the conjugate remains almost unchanged, with only minor shifts of 0.01 to 0.02 ppm. These shifts suggest the presence of non-covalent interactions and slight changes in electron distribution but do not result in significant alterations to the protein structure.

#### 2.2.2. FT-IR Spectroscopy

**Figure 3:**
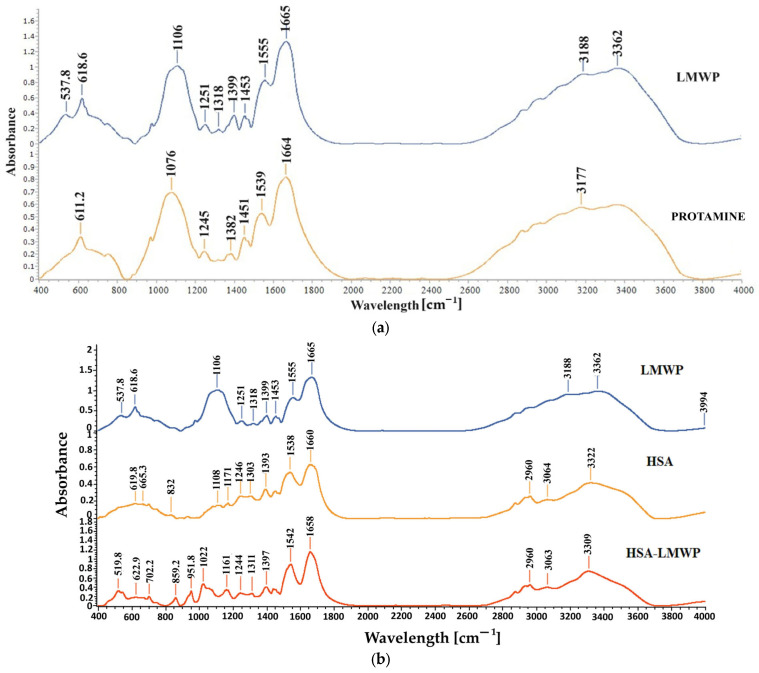
FT-IR spectra recorded for protamine and LMWP (**a**), as well as for LMWP, HSA, and HSA modified with LMWP (**b**).

FT-IR spectra were obtained for standard protamine, LMWP, HSA, and LMWP-modified HSA to support the structural analysis. These spectra are presented in Figure 3 and provide additional information on the functional interactions between the compounds.

The amide I band, which ranges from 1600 to 1700 cm^−1^, is the most commonly used feature in studies of protein secondary structure, particularly for proteins and peptides. In the spectra of protamine and LMWP, as shown in Figure 3a, a peak is observed at 1665 cm^−1^ for protamine and at 1664 cm^−1^ for LMWP, and the peak is associated with the C=O stretching vibration that is observed in conjunction with the NH of the peptide bond. Additionally, the amide II peak in the protamine spectrum is noted at 1539 cm^−1^, while in the LMWP spectrum, this peak shifts to 1555 cm^−1^. The peak intensity in the LMWP spectrum has shifted slightly by 16 cm^−1^. This change may indicate a transformation in the secondary structure of protamine, possibly from an α-helix to a β-sheet, or it could suggest the formation of new hydrogen bonds following the cleavage of protamine. In the region between 400 and 1500 cm^−1^, the enhancement of the band at 1106 cm^−1^ indicates the C–N stretching of the guanidinium groups from arginine, which constitutes approximately 67% of the total content and is more exposed in the LMWP spectrum [70]. In the spectrum of LMWP, an absorption peak is observed at 537.8 cm^−1^. This peak can be attributed to either NH vibrations or C-N/C-C bonds within the polypeptide chain. These observations confirm that LMWP possesses a shorter peptide structure with exposed functional groups and a slight conformational rearrangement compared to native protamine.

Figure 3b shows the FT-IR spectra for LMWP, HSA, and HSA modified with LMWP. In the spectrum of native albumin (Figure 3b), a broad N–H/O–H stretching band appears at 3322 cm^−1^. This band shifts slightly to 3309 cm^−1^ in the spectrum of LMWP-functionalized HSA, indicating a change in hydrogen bonding interactions, possibly due to interactions with arginine groups. The amide I band, corresponding to the α-helix secondary structure of the protein, is observed at approximately 1660 cm^−1^, while the amide II band appears at 1538 cm^−1^ in the HSA spectrum. Following functionalization with LMWP, these bands exhibit a slight shift, suggesting possible conformational changes or the introduction of new intermolecular interactions.

The amide III band, observed at 1246 cm^−1^ in native HSA, appears at 1244 cm^−1^ in HSA–LMWP with lower intensity, which could suggest intermolecular interactions such as hydrogen bonding. The absorption peak at 1022 cm^−1^ is characteristic of aliphatic C–N stretches, attributed to arginine and lysine residues [71].

The peak at 951.8 cm^−1^ suggests the presence of aliphatic CH groups or N–H bending vibrations, which are typical of amines, indicating that amino acid side chains are present in LMWP. The peak at 859 cm^−1^ corresponds to C–C–N or C–C–C bending vibrations, characteristic of amino acids such as proline and glycine [72]. Additionally, signals indicating the formation of thioether (C–S) bonds may also be observed in this region, since the coupling agent SMCC was used to conjugate Cys34 of HSA with LMWP.

The peak at 519.8 cm^−1^ is attributed to C–S stretching vibrations, which are characteristic of thioether bonds or possibly disulfide (S–S) bridges [72,73]. This signal confirms the formation of a covalent bond between the maleimide group of SMCC and the thiol of Cys34 in albumin, indicating the successful conjugation reaction with LMWP. Additionally, these new bands, which are absent in the spectrum of native HSA, support the formation of a stable thioether-type covalent bond between LMWP and HSA in the presence of SMCC.

### 2.3. Obtaining and Characterization of the Nanoparticles

#### 2.3.1. Obtaining Nanoparticles with and Without Encapsulated Drugs Through the Self-Assembly Method

**Figure 4:**
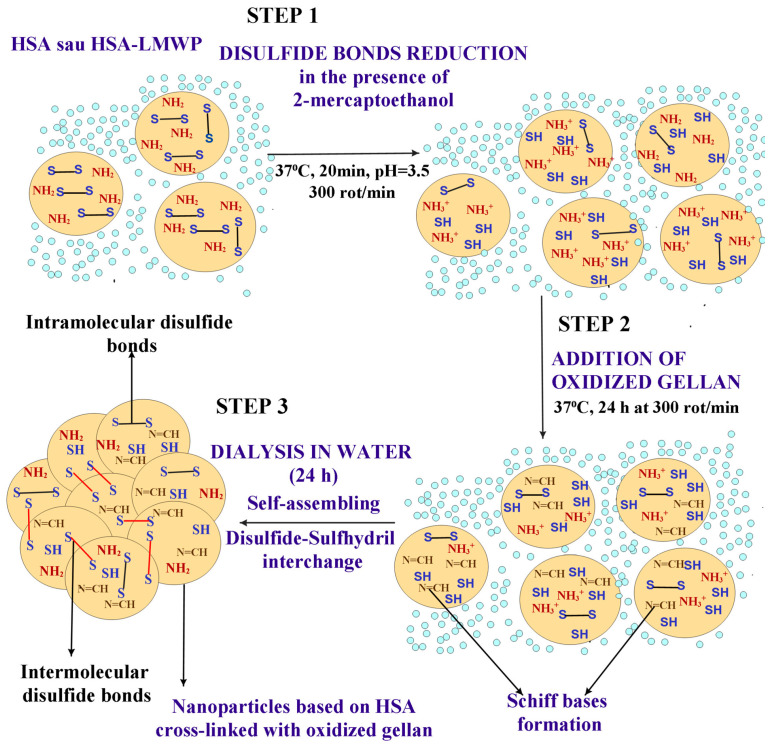
Mechanism for obtaining albumin nanoparticles crosslinked with oxidized gellan through self-assembly.

Figure 4 illustrates a schematic representation of the nanoparticle formation mechanism. The physicochemical properties of the nanoparticles are influenced by the molar ratio between the amine groups in albumin and the aldehyde groups in OG, as well as the type of drug used.

The molar ratios of amine groups in albumin to aldehyde groups in the OG studied were 1:0, 1:0.25, 1:0.5, 1:0.75, 1:1, 1:1.5, and 1:2. The particle sizes ranged from 68 nm to 380 nm, depending on the molar ratio employed. To achieve optimal particle sizes of up to 200 nm, the molar ratios of 1:0, 1:0.5, 1:0.75, and 1:1 were utilized. Albumin nanoparticles, whether uncrosslinked or crosslinked with OG, were developed to encapsulate TMZ, curcumin, or a combination of both through self-assembly. This technique allows for the formation of albumin nanoparticles by incorporating β-mercaptoethanol, which enhances the protein’s hydrophobicity by breaking disulfide bonds. Alternatively, the protein’s surface amine groups can be modified by attaching a hydrophobic (lipophilic) compound to improve their properties [37].

^1^H NMR and FT-IR spectroscopy confirmed the successful synthesis of LMWP and demonstrated that albumin was modified with LMWP at the thiol group in its structure.

The experimental program is provided in Table 2.

Albumin nanoparticles can be obtained using the self-assembly technique with β-mercaptoethanol to enhance the hydrophobicity of the protein by cleaving disulfide bonds or by reducing the amine groups on the protein surface through conjugation with a hydrophobic (lipophilic) compound [37,74,75]. Albumin self-assembly can occur when the unfolding of the protein is triggered by the reduction of disulfide bonds, typically using agents such as β-mercaptoethanol. Following this, the addition of a hydrophobic drug results in the self-assembly of albumin into nanoparticles. This results from non-covalent hydrophobic interactions between the drug and the albumin [37]. Subsequently, glutathione (GSH), a reducing agent, was introduced into the modified human serum albumin (HSA) at 37 °C, exposing the albumin’s hydrophobic binding domains. Excess GSH was removed through dialysis, after which an ethanolic solution of PTX was added to form the nanoparticles. The resulting redox-sensitive delivery systems were formed through the intermolecular disulfide bridges that developed during self-assembly [76]. PEGylated albumin was conjugated with a W peptide (Trp-Lys-Tyr-Met-Val-D-Met) designed to target the formyl peptide receptor, which is overexpressed in triple-negative breast cancer (TNBC). This modified albumin was then used to prepare nanoparticles loaded with Paclitaxel (PTX).

To achieve this, glutathione (GSH), a reducing agent, was introduced into the modified HSA at 37 °C, exposing the hydrophobic binding domains of the albumin. Excess GSH was subsequently removed through dialysis. After this, an ethanolic solution of PTX was added to develop the nanoparticles. The resulting redox-sensitive delivery systems are formed due to the intermolecular disulfide bridges created during self-assembly [77]. Researchers have found that a pH value of 9 has a negative impact on the self-assembly of nanoparticles. This results from the enhanced electrostatic repulsion between albumin macromolecules, which decreases the protein’s capacity to form nanoparticles through self-assembly [77,78]. Previous research [79] has indicated that the optimal pH for protein self-assembly lies between 7 and 8 [79]; yet, at this pH, curcumin and TMZ are easily degraded [80,81]. We selected a pH of 3.5 for this study because it is the optimal point at which both curcumin and TMZ remain stable. At this pH, the amine groups become protonated, allowing them to interact with the OG, which serves as a crosslinking agent to enhance the stability of the nanoparticles. To avoid significantly increasing the particles’ hydrophilicity and size, we limited the molar ratios of NH_2_ groups in HSA to aldehyde groups in OG to 1:0.5, 1:0.75, and 1:1.

#### 2.3.2. Determination of the Mean Particle Size

The average diameter of nanoparticles containing immobilized drugs and their size distribution were examined using laser beam diffractometry with the SHIMADZU-SALD 7001 diffractometer (Shimadzu Corporation, Kyoto, Japan). Table 3 presents the average diameter (D50) of the particles synthesized through self-assembly. Additionally, Table S1 in the Supplementary Materials presents the values D25, D50, and D75 for the obtained samples. Figure S3 shows the polydispersity curves for samples prepared with unmodified albumin.

It is observed that the size of the nanoparticles increases with the amount of OG, thereby increasing the degree of crosslinking. For the non-crosslinked nanoparticles, it is noted that in some cases, such as samples NTC0 and MT0, the size is larger than that of NTCG1 and MTG2 due to a greater amount of encapsulated drug.

Nanoparticles modified with LMWP display a smaller size than unmodified nanoparticles (Table 3, Table S1, and Figure S3), likely due to structural changes in albumin following functionalization [28]. Most nanoparticles measure less than 150 nm, allowing them to effectively serve as drug delivery systems that can overcome the BBB. The literature indicates that a key feature of the EPR effect is the highly permeable tumor vasculature, which enhances the permeability of particles in the 20–200 nm size range [82].

The average diameter of the HSA-based nanoparticles obtained in this study, ranging from 67 to 145.3 nm, as determined by SALD, is consistent with the sizes reported for albumin nanoparticles intended to cross the BBB. The literature suggests that sizes between 10 and 100 nm are optimal for effectively targeting the BBB [83]. Increased crosslinking in albumin nanoparticles results in a notable increase in their size, reaching approximately 292.3 nm at an NH_2_:CHO molar ratio of 1:1. This suggests that larger protein aggregates are formed, consistent with the observed increase in size. This observation is consistent with the existing literature, which indicates that the average size of albumin nanoparticles, typically around 100 nm, can increase when either the amount of albumin or the concentration of the crosslinking agent increases [25]. Glutaraldehyde-crosslinked albumin nanoparticles usually have an average diameter ranging from 100 to 150 nm [84]. However, nanoparticles with a higher degree of crosslinking can reach diameters of 281 nm, which exceeds the optimal range for overcoming the BBB.

Furthermore, the preparation conditions, such as the pH of the albumin solution, significantly influence the final particle size. For example, as the pH increases, the diameter of the particles decreases due to enhanced ionization and electrostatic repulsion between HSA molecules [85]. Therefore, developing albumin nanoparticles with a diameter below 150 nm is essential to ensure the biodistribution and permeability necessary to overcome the BBB [85].

The modification of albumin using the cationic peptide LMWP produced particles with smaller diameters compared to unmodified albumin. Other researchers have also noted this beneficial result. For example, nanoparticles based on BSA that were functionalized with LMWP were found to have dimensions of less than 150 nm [25]. Utilizing a modified HSA for nanoparticle synthesis resulted in sizes remaining within the nano range, suggesting that functionalizing with peptides to improve cellular diffusion can prevent considerable increases in nanoparticle size and enhance permeability across the BBB [25].

HSA nanoparticles, enhanced with peptides such as LMWP to promote intracellular diffusion, typically measure between 100 and 130 nm in size. In systems containing hydrophobic drugs such as paclitaxel and fenretinide, the successful co-encapsulation of these active compounds was achieved in nanoparticles approximately 100 nm in size. Incorporating peptides to enhance cellular diffusion (such as LMWP) facilitated effective passage across the BBB through biomimetic approaches. Delivery strategies at the cellular level or within the central nervous system include receptor-mediated transport, non-specific adsorption followed by endocytosis, passive diffusion, and peptide-assisted direct internalization, all of which aim to enhance the diffusion of nanoparticles within the cells. Moreover, specific nanoparticles are designed to mimic the structure of viruses or lipoproteins, possessing protein shells that cells in the BBB or central nervous system naturally recognize [85]. HSA nanoparticles conjugated with specific transport ligands, such as apolipoprotein E (ApoE), typically have a size range of 120–150 nm. A study by Zensi et al. demonstrated that HSA nanoparticles that were covalently modified with ApoE were internalized by brain endothelial cells and successfully reached neurons.

In contrast, unmodified nanoparticles failed to diffuse through the brain tissue. Similarly, Ulbrich et al. showed that HSA nanoparticles modified with transferrin or anti-transferrin receptor antibodies effectively transported drug molecules, specifically, loperamide, across the BBB. These findings highlight the effectiveness of functionalizing nanoparticles with specific ligands. The rise in electrostatic repulsion among albumin molecules, due to increased ionization at higher pH values, limits their agglomeration and, consequently, leads to the formation of smaller nanoparticles [86]. At higher pH levels, albumin becomes more ionized, which enhances particle stabilization through increased electrostatic repulsion. This characteristic is essential for maintaining a compact and stable nanoparticle structure, which is crucial for successfully crossing the BBB. Overall, research indicates that albumin nanoparticles between 80 and 150 nm can effectively penetrate brain tissue by utilizing biomimetic transport mechanisms, including interactions with albumin-binding proteins or lipoprotein receptors [25]. Functionalization with peptides to enhance cellular diffusion (LMWP, etc.) or ligands such as ApoE or transferrin has facilitated the development of HSA/BSA particles measuring approximately 100–150 nm that effectively overcome the BBB [87]. Particles larger than 200 nm in diameter are considered ineffective because they are rapidly removed from the bloodstream by peripheral organs, such as the liver and spleen, through the reticuloendothelial system, significantly reducing their chances of reaching the central nervous system [25]. These data highlight the significance of size control and electrostatic interactions in designing albumin nanoparticles for the treatment of brain diseases [85].

The size distribution of nanoparticles was assessed using the D25%, D50%, and D75% values. The polydispersity determination of the samples was expressed by the SPAN index, calculated according to the relationship presented in the Section 4 [88]. The SPAN values obtained, which range from 0.26 to 0.56, suggest that the nanoparticles obtained have a uniform and precise size distribution. Most of the nanoparticles exhibit a SPAN value of less than 0.45, indicating low and homogeneous polydispersity in particle sizes. The lowest value recorded for the NCG3 nanoparticles (SPAN = 0.26) signifies a highly uniform size distribution, indicating excellent reproducibility and colloidal stability.

In contrast, the NTCG2 formulation, which has a SPAN of 0.56, exhibits a broader dispersion, implying a somewhat higher level of polydispersity. This difference may be attributed to the potential for nanoparticle aggregation, which could affect the controlled release of the active principles and their long-term stability. In the literature, it is considered that low values of SPAN are essential for obtaining uniform nanoparticles with predictable behavior and improved stability in pharmacological and biotechnological applications [88].

#### 2.3.3. Scanning Electron Microscopy

**Figure 5:**
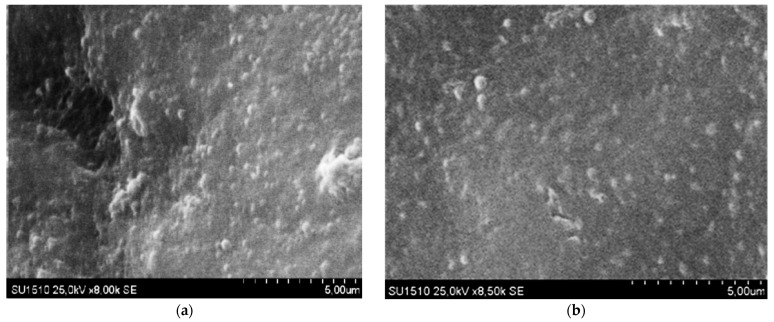
Scanning electron microscopy images for MTCG2 nanoparticles recorded from different perspectives: (**a**) micrograph obtained from one viewing angle; (**b**) micrograph of the same sample from an alternative angle.

Scanning electron microscopy (SEM) was used to collect data on the morphology of nanoparticles containing encapsulated drugs. Figure 5 displays the SEM images of the MCTG2 samples, providing details about the particle size and shape.

A high number of nanoparticles are observed in Figure 5, which tend to aggregate in the dry state due to the lyophilization process. This behavior is common in albumin-based delivery systems and aligns with data from the literature, where similar results have been reported for BSA particles with encapsulated curcumin [89].

#### 2.3.4. FT-IR Spectroscopy of Nanoparticles

**Figure 6:**
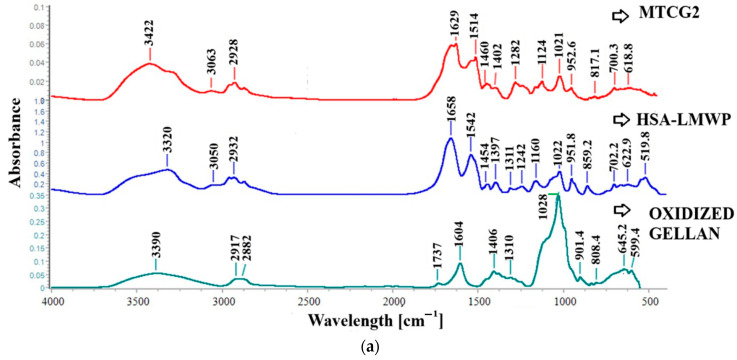

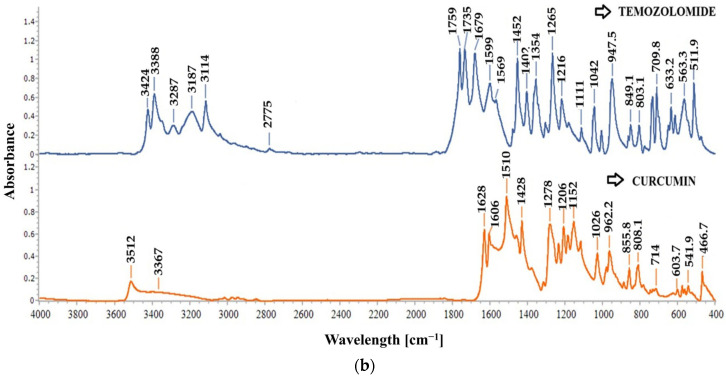
FTIR spectra of particles obtained by MTCG2 self-assembly for HSA modified with LMWP and OG (**a**) and FT-IR spectra of the two drugs encapsulated in nanoparticles, temozolomide and curcumin, respectively (**b**).

The FT-IR spectra shown in Figure 6 illustrate the structural changes observed after crosslinking albumin nanoparticles with oxidized gellan (OG) through Schiff base formation, enabling the identification of the involved functional groups.

Albumin shows the amide bands typical of proteins, and the absorption peak of the amide band I, at 1658 cm^−1^, results from C=O stretching vibrations, while the amide group II band at 1542 cm^−1^ is due to C–N stretching vibrations and N–H bending vibrations. Regarding HSA nanoparticles crosslinked with OG (MTCG2), the stronger absorption band at 1629 cm^−1^ and the shoulder at 1660 cm^−1^ are linked to the C=N stretching vibrations of the Schiff bases and the C=O stretching vibrations of unreacted carbonyl groups. The Schiff bases are formed through the crosslinking reaction between albumin’s amine groups and the carbonyl groups of OG. The peak at 1514 cm^−1^ in the nanoparticle spectrum indicates an amide band shift that becomes more noticeable after the crosslinking process.

The characteristic peaks of OG are present in the spectrum of the analyzed samples; however, they are slightly shifted in wavelength. Notably, the peak associated with the aldehyde group, which appears in the spectrum of OG at 1737 cm^−1^, is absent in the spectrum of MTCG2 nanoparticles. This disappearance of the peak linked to the carbonyl group may indicate that the aldehyde groups were consumed during the crosslinking reaction with the amine groups. Additionally, it suggests that TMZ could have been conjugated to the amine groups of the LMWP-modified albumin. In the nanoparticle spectrum, peaks characteristic of the drugs are observed at slightly shifted wavelengths with reduced intensity, indicating their encapsulation within the nanoparticles obtained. The FT-IR spectra (Figure 6) confirm the absence of a peak characteristic of the aldehyde group in the OG within the MTCG2 nanoparticle spectrum, supporting the hypothesis that crosslinking occurs with amine groups.

Additionally, the peaks corresponding to the drugs appear at slightly shifted wavelengths and with reduced intensity, indicating that they are encapsulated within the nanoparticles. TMZ was effectively encapsulated in a polymer matrix formed by albumin modified with low-molecular-weight protamine (LMWP) and crosslinked with oxidized gellan (OG). The cleavage of disulfide bridges in the presence of mercaptoethanol exposed the hydrophobic domains of albumin, facilitating stable non-covalent interactions with both TMZ and curcumin, likely through hydrogen bonds and hydrophobic interactions. The observed shifts and decreases in the bands associated with the active compounds in the FT-IR spectra suggest effective encapsulation. In the case of TMZ, there may also be an association with the exposed amino groups of protamine.

#### 2.3.5. Evaluation of the Drug Encapsulation Efficiency

The immobilization efficiency of curcumin and temozolomide in the self-assembled nanoparticles was evaluated as an indicator of the encapsulation system’s performance. Table 4 presents the results obtained from this analysis.

According to the data in Table 4, curcumin shows higher immobilization efficiency than TMZ, regardless of the type of nanoparticles analyzed. This difference can be attributed to the presence of a free amine group in the structure of TMZ. This amine group can react with carbonyl groups that remain unreacted with the amine groups in HSA, forming a polymer–drug conjugate. As a result, this interaction reduces the amount of TMZ that can be extracted in ethanol, contributing to the lower immobilization efficiency observed. Helal reported similar findings to those of D.O. et al. [90], who achieved an encapsulation efficiency of approximately 1.5%. They attributed this low value to the instability of TMZ in ethanol. In our study, although the drug was initially solubilized in DMSO for nanoparticle formulation, ethanol was later used to evaluate the encapsulation efficiency. This switch may have contributed to the partial degradation of TMZ.

Furthermore, it is noted that the drug immobilization efficiency in low-molecular-weight protamine (LMWP)-modified HSA nanoparticles is comparable to that of unmodified particles, and in some cases, it is even superior. This enhancement may be due to the presence of a greater number of free amine groups in the modified protein structure, which facilitates interaction with active molecules. Curcumin possesses a planar, polyphenolic, and hydrophobic structure, enabling it to bind effectively to various hydrophobic sites on albumin. This strong binding results from hydrophobic interactions, π–π stacking facilitated by the planar surfaces of aromatic nuclei, and hydrogen bonding. Consequently, curcumin can reach up to 8.33% (*w*/*w*) loading capacities in self-assembled albumin nanoparticles [91,92]. In comparison, TMZ, a smaller polar molecule that tends to undergo hydrolytic degradation at physiological pH, interacts less effectively with the protein matrix. Under optimized conditions, TMZ achieves a loading capacity of approximately 5.5% (*w*/*w*) in albumin nanoparticles [90].

The encapsulation efficiency (EE) of curcumin in HSA nanoparticles ranges from 77% at a lower concentration of 10 µg/mL to 93% at higher concentrations due to strong hydrophobic interactions and the affinity for albumin’s hydrophobic domains [93]. The literature reports indicate that the immobilization efficiencies of TMZ in albumin nanoparticles range from 15% to 35%. For instance, Helal et al. enhanced the loading of TMZ acid (TMZA) within HSA-based nanoparticles, achieving encapsulation efficiencies that depend on the pH of the desolvation medium and the TMZA: albumin ratio, with values ranging from 15.2% to 33.8% [90]. Kudarha and Sawant developed chondroitin sulfate-conjugated BSA nanoparticles (CS-TNPs) and reported an encapsulation efficiency (EE) of approximately 32% ± 2% [91]. These results align closely with the 15–34% range observed in the experiments conducted in this study, which used 10 mg of TMZ in 20 mL, demonstrating the method’s consistency and reproducibility.

#### 2.3.6. Study of the Release Kinetics of Curcumin and TMZ from Nanoparticles

**Figure 7:**
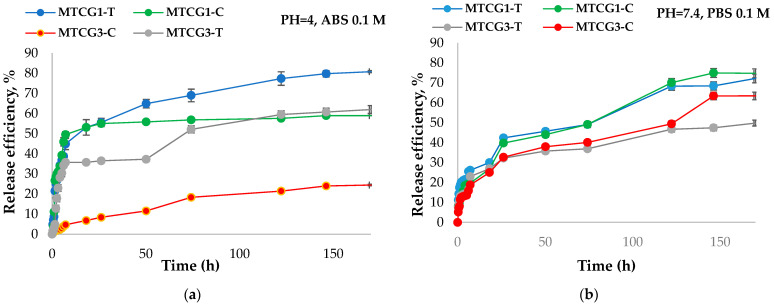

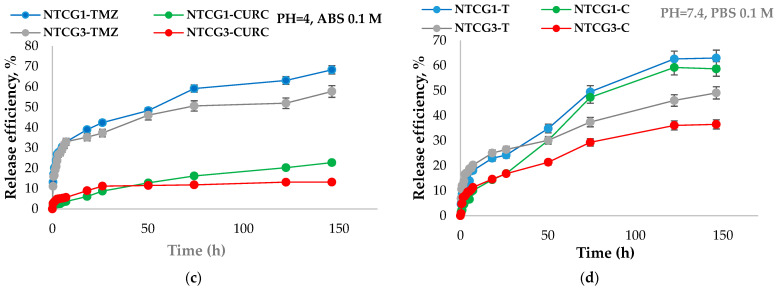
Release kinetics of curcumin and TMZ (in terms of efficiency) over time from LMWP-modified HSA-based nanoparticles crosslinked with OG: in ABS, 0.1 M, at pH 4 (**a**); in PBS at pH 7.4 (**b**); and from unmodified HSA-based nanoparticles crosslinked with OG: in ABS, 0.1 M, at pH 4 (**c**); and PBS at pH 7.4 (**d**).

The release kinetics of curcumin and TMZ from two types of HSA nanoparticles—one functionalized with LMWP and the other unfunctionalized—were studied at a physiological temperature of 37 °C in two different pH environments containing 1% (*w*/*w*) Tween 80. These environments consisted of a 0.1 M phosphate buffer solution (PBS) at pH 7.4 and a 0.1 M acetate buffer solution (ABS) at pH 4. At predetermined time intervals, samples were collected, and the absorbance was measured at 425 nm for curcumin and 327 nm for TMZ. Based on the calibration curve equations presented in Section 4 (Methods), the amount of active compound released was determined. Figure 7 shows the kinetic curves for the release of curcumin from the tested samples. While the release kinetics study covered most samples, we will specifically highlight the following curves: those for curcumin and TMZ co-encapsulated in LMWP-functionalized HSA nanoparticles crosslinked with OG (MTCG1 and MTCG3) and for the unmodified HSA nanoparticles (NTCG1 and NTCG3).

Research has shown that the pH of the release medium influences release efficiency. Specifically, the efficiency of curcumin release from nanoparticles is higher at a pH of 7.4 compared to a pH of 4. In contrast, the release of TMZ from HSA nanoparticles is more efficient at pH 4 than at pH 7.4. The Ritger–Peppas model [94] was applied to better understand the drug release mechanism from the polymer matrix. Table 5 presents the immobilization efficiency and the exponential factor n determined based on the Ritger–Peppas equation. At pH 4, the release mechanism for curcumin and TMZ from samples NTCG1, NTCG2, and NTCG3 is identified as Fickian, although it is affected by potential interactions between the drug and the biopolymers, such as hydrophobic–hydrophobic ones (*n* < 0.5). In contrast, with the LMWP-modified albumin samples MTCG1, MTCG2, and MTCG3, complete drug release is achieved in MTCG1 at pH 4, where n values are 1 for curcumin and 0.87 for TMZ, indicating a diffusion case II. However, for the drugs released from MTG2 and MTCG3 at pH 4, n decreases, implying a non-Fickian diffusion mechanism for the release of curcumin (from sample MTCG3) and TMZ (from both samples), influenced by both diffusion and matrix erosion.

The release mechanism of curcumin from the MTCG2 sample is characterized as Fickian and is primarily driven by diffusion. At pH 7.4, the release of TMZ from all examined samples also demonstrates a Fickian or semi-Fickian diffusion mechanism. A similar mechanism was observed for the release of curcumin from LMWP-functionalized samples (MTCG1, MTCG2, MTCG3) at pH 7.4, where the value of n is approximately 0.3, suggesting the presence of hydrophobic interactions between curcumin and the hydrophobic domains in albumin. In contrast, the unmodified samples NTCG1, NTCG2, and NTCG3 at pH 7.4 exhibit n values greater than 0.5, indicating a non-Fickian diffusion mechanism influenced by both diffusion and degradation processes.

The release kinetics of curcumin and TMZ from nanoparticles (Figure 7) indicate that the surrounding medium’s pH influences the release efficiency. Notably, the release efficiency of curcumin from nanoparticles at pH 7.4 is higher than that observed at pH 4. This difference can be attributed to two main factors: the increased solubility of curcumin in slightly alkaline conditions and the deprotonation of free functional groups in the OG, which converts them to carboxylate groups. This conversion enables the relaxation of the polymer network, thereby facilitating greater diffusion of the encapsulated active substances. In contrast, the release of TMZ from HSA-based nanoparticles is more efficient at pH 4 than at pH 7.4. The release efficiency of TMZ may be enhanced because, at this acidic pH, albumin undergoes acid hydrolysis [95], which leads to weakening of the polymer network and the release of a larger amount of TMZ, which is more soluble at this pH compared with curcumin, which is soluble in an alkaline medium. The release of active compounds is influenced by the degree of crosslinking of the nanoparticles. It has been observed that the release efficiency decreases as the degree of crosslinking increases. The crosslinking degree is higher when there is an increased molar ratio of aldehyde groups in OG to amine groups in HSA. For active compounds that are co-encapsulated in HSA nanoparticles functionalized with LMWP obtained through self-assembly, the release efficiency of both TMZ and curcumin is greater compared to the release efficiency of these compounds when they were co-encapsulated in unmodified HSA-based nanoparticles.

The exponential factor n (see Table 5) indicates that at a pH of 4, the diffusion mechanism for curcumin and TMZ released from the unmodified LMWP samples is Fickian. However, this mechanism is influenced by various interactions that may take place between the drugs and the biopolymers. These interactions are likely stronger for curcumin, which exhibits a release efficiency ranging from 13.16% to 22.76%. The exponential factor n for curcumin falls between 0.245 and 0.298. In contrast, TMZ has a release efficiency ranging from 57.71% to 68.36%, with the exponential factor n varying from 0.35 to 0.402.

At pH 4, the amino groups in albumin are likely to be protonated because this pH value is lower than the pKa of albumin. This protonation allows them to interact with drugs, particularly curcumin, by forming hydrogen bonds. At acidic pH levels, these bonds are stronger and less likely to dissociate. In the analyses of samples containing modified albumin, we observed that the release of drugs is complete for MTCG1 at pH 4. The exponential factor n was 1 for curcumin and 0.87 for TMZ, indicating that for this sample, there is a possibility that the nanoparticles may degrade (release mechanism governed by matrix erosion), as albumin is known to undergo acid hydrolysis at acidic pH [96]. The study on the release kinetics was conducted over approximately one week. We observed that for the drugs released from samples MTCG2 and MTCG3 at pH 4, the exponential factor n decreased, indicating a non-Fickian diffusion mechanism for releasing curcumin from sample MTCG3 and TMZ from both samples. This mechanism is influenced by both diffusion and degradation.

In contrast, the release of curcumin from sample MTCG2 follows a Fickian mechanism governed solely by diffusion. At pH 7.4, all analyzed samples exhibit a Fickian or semi-Fickian diffusion mechanism for TMZ release, likely influenced by interactions between the drug and functional groups in albumin. A similar diffusion mechanism was observed in the release of curcumin from functionalized samples with LMWP (MTCG1, MTCG2, MTCG3) at pH 7.4, where the exponential factor n is around 0.3, probably suggesting hydrophobic interactions between curcumin and the hydrophobic regions of albumin.

When comparing the release efficiencies of curcumin and temozolomide (TMZ) from the same type of nanoparticles at the same pH, we observed statistically significant differences, particularly at pH 4. At pH 7.4, significant differences in release efficiencies between TMZ and curcumin were found only for the NTCG2 nanoparticles (*p* = 0.04), while there was a trend for NTCG3 (*p* = 0.06) and a statistically significant difference for MTCG3 (*p* = 0.0022). The effect of pH (4 vs. 7.4) on the release efficiency of TMZ and curcumin from the same type of samples was examined. It was found that for TMZ, the differences in release efficiencies from nanoparticles at pH 4 compared to pH 7.4 vary based on the formulation of the nanoparticles and are statistically significant for NTCG3 (*p* = 0.019), MTCG2 (*p* = 0.01), and MTCG3 (*p* = 0.002). In the case of curcumin, the differences in release efficiency from nanoparticles at pH 4 versus pH 7.4 are statistically significant across all samples, with *p*-values ranging from 0.003 to below 0.0001.

The statistical analysis investigated how functionalizing nanoparticles with LMWP affects the release kinetics of active compounds from both functionalized and non-functionalized samples at the same pH level.

At pH 4, the release of TMZ from various nanoparticles showed statistically significant differences in release efficiency for the NTCG1/MTCG1 samples (*p* = 0.00101) and NTCG3/MTCG3 samples (*p* = 0.0404). However, there was no significant difference observed for the NTCG2/MTCG2 samples (*p* = 0.77). Regarding curcumin release at pH 4, the differences in release efficiency between unfunctionalized and functionalized nanoparticles were statistically significant across all formulations (*p* < 0.0001), with the NTCG1/MTCG1 samples exhibiting a relatively moderate difference (*p* = 0.003).

At pH 7.4, the release efficiencies of TMZ revealed significant differences for the NTCG1/MTCG1 samples (*p* = 0.03) and NTCG2/MTCG2 samples (*p* = 0.008), while the NTCG3/MTCG3 samples showed no statistically significant difference (*p* = 0.23). For curcumin release efficiency, significant differences were observed in the NTCG1/MTCG1 samples (*p* = 0.015) and NTCG3/MTCG3 samples (*p* < 0.0001), while the NTCG2/MTCG2 samples did not show a significant statistical difference (*p* = 0.2).

Statistical analysis revealed significant differences in the release profiles of curcumin and TMZ from nanoparticles at pH 4. However, at pH 7.4, these differences diminish and are influenced by factors such as the degree of crosslinking of the nanoparticles or their functionalization with LMWP. In summary, at pH 4, the differences in release efficiency between curcumin and TMZ are statistically significant; however, at pH 7.4, the release efficiencies become less distinct and remain dependent on the formulation of the nanoparticles.

The release of curcumin and temozolomide is influenced by the degree of crosslinking (molar ratio) and the presence of LMWP. The results show a clear impact of LMWP on the release efficiency of curcumin, with significant statistical differences observed across all comparisons. In contrast, the effect of LMWP on temozolomide is noticeable only in specific combinations of molar ratio and pH. By adjusting the degree of crosslinking and/or the functionalization with LMWP, it is possible to fine-tune the kinetics of the release process.

### 2.4. Biological Properties and Functional Evaluation of the Functionalized Albumin Nanoparticles

#### 2.4.1. Evaluating Cell Apoptosis Induced by Drug-Loaded Nanoparticles

**Figure 8:**
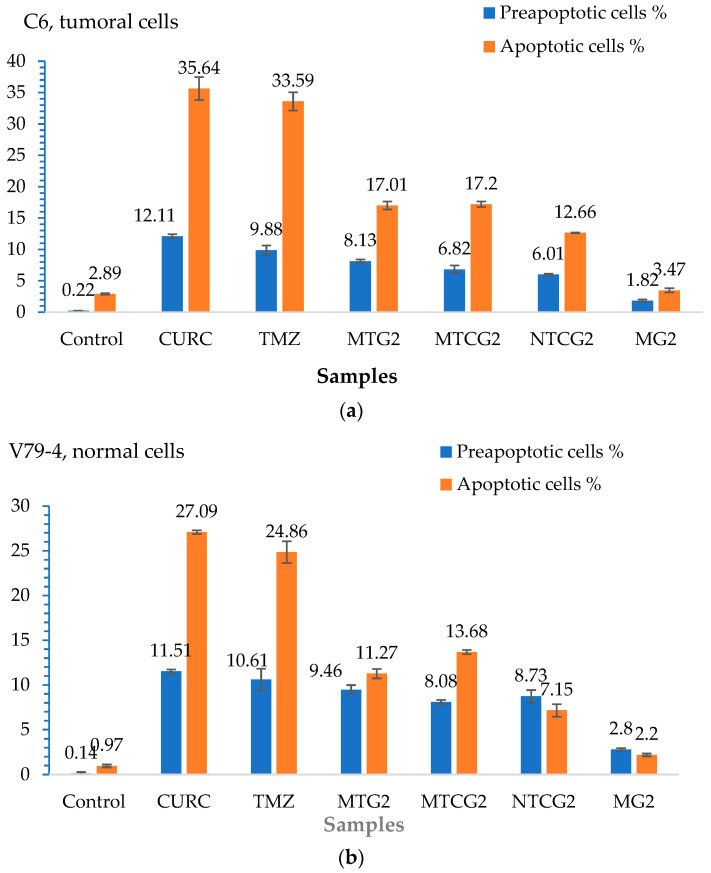
Effect of different types of nanoparticles on the percentage of pre- and apoptotic cells in the C6 (**a**) and V79-4 (**b**) cell lines (*p* < 0.05).

The possible mechanism of action for the analyzed nanoparticles was established by determining the process of cellular apoptosis using flow cytometry, a method that enabled the assessment of the percentage of live, dead, pre-apoptotic, and apoptotic cells (Table S2 and Figure 8). The analysis of pre-apoptotic cell percentages in the C6 tumor cell line reveals a significant increase following treatment with standard drugs, specifically, 12.11% for curcumin (CURC) and 9.88% for TMZ. Additionally, albumin nanoparticles crosslinked with OG increased the number of early apoptotic cells, with rates of 8.13% for MTG2, 6.82% for MTCG2, and 6.01% for NTCG2. Furthermore, the frequency of apoptotic cells increased with curcumin treatment to 35.64% and with TMZ to 33.59%. In contrast, the application of MTG2 (17.1%) and MTCG2 (17.2%) nanoparticles yielded lower values than free drugs, which can be attributed to the reduced amount of encapsulated drug.

In the case of the normal cell line V79-4, a response comparable to that obtained in the tumor line (CURC–11.51% and 10.61%–TMZ) was noted in terms of the frequency of pre-apoptotic cells for the chemotherapeutics studied. The proportion of pre- and apoptotic cells in the normal cell line V79-4 is similar in value, especially with nanoparticles containing both drugs, suggesting a synergistic effect between them (Figure 8b).

The differences in pre-apoptotic cells between the C6 tumor cell line and the V79-4 cell line, both treated with different types of nanoparticles, were statistically significant, with a *p*-value less than 0.05. For the same cell lines treated with free drugs, there were no statistically significant differences in the percentage of pre-apoptotic cells, with a *p*-value greater than 0.05. However, statistically significant differences were observed in the percentage of apoptotic cells between the normal cell line and the tumor cell line treated with nanoparticles or free drugs, with a *p*-value of less than 0.05.

The difference in the percentage of pre-apoptotic cells within the C6 tumor line caused by free drugs (CURC–12.11% and 9.88%–TMZ) compared to encapsulated drugs, 8.13% for MTG2 (*p* = 0.05), 6.82% for MTCG2 (*p* = 0.01), and 6.01% for NTCG2 (*p* = 0.01), is due to a lower amount of drug being trapped within the particles. This study focused on the concentration of nanoparticles, without considering the quantity of the included drug. For example, in the MTCG2 sample, the total drug content within 7.5 mg of nanoparticles/g is 0.73 µg/mL (0.6 µg of curcumin and 0.12 µg of TMZ), while the MTG2 sample contains 0.17 µg/mL of TMZ. In comparison, free drugs reach a concentration of 7.5 µg/mL, providing context for the observed results. The results indicate that nanoparticles can induce pre-apoptosis or cellular apoptosis, even when the amount of encapsulated drug is significantly lower than that of the free drug (at least 20 times lower in the case of curcumin). This response may imply the presence of a similar mechanism, as it increases the levels of pre-apoptotic proteins (caspase-3 and Bax), which play a role in initiating programmed cell death [97,98,99]. Apoptosis in the normal cell line V79-4 (Figure 8b) is lower than in the cancer cell line C6. A dose–effect relationship was observed for both cell lines, and all types of nanoparticles were tested. The intensity of the apoptosis process varies between different types of nanoparticles. The combination of the two drugs induces a synergistic effect, resulting in a stronger response from tumor cells than normal cells.

To assess apoptosis, we used Annexin V-FITC/PI as a blank control, which allowed for clear differentiation between viable, pre-apoptotic, and apoptotic cells, ensuring the validity of the results obtained from flow cytometry. As a positive control, we employed the MG2 sample, which consists of empty nanoparticles functionalized with LMWP and without any drug, to verify the potential impact of the matrix on cell viability.

To highlight the role of LMWP functionalization in the pro-apoptotic effects of nanoparticles, a comparison was made between functionalized samples (MTG2 and MTCG2) and non-functionalized ones (NTCG2), which contained either TMZ or TMZ combined with curcumin immobilized. An increase in the percentage of cells in pre-apoptosis is observed with functionalized nanoparticles: 8.13% for MTG2 and 6.82% for MTCG2, compared to 6.01% for NTCG2, using the C6 tumor cell line. Although the differences are not substantial, they can be attributed to the ability of LMWP to enhance cellular diffusion, which results in a more effective release of active substances at the intracellular level. The larger difference seen with MTG2, compared to the other two samples, is due to it containing double the amount of TMZ compared to NTCG2 and MTCG2 (only the concentration of nanoparticles was considered in this study, and not the drug concentration). The differences in the percentage of pre-apoptotic cells after treating C6 cells with MTG2 nanoparticles compared to treatments with MTCG2 and NTCG2 samples are statistically significant, with a *p*-value less than or equal to 0.05.

In the case of the C6 tumor cell line, the percentages of pre-apoptotic and apoptotic cells were significantly higher after treatment with nanoparticles loaded with active substances (MTG2, MTCG2, NTCG2) compared to those treated with MG2, confirming the crucial role of the drug substances in inducing apoptosis (*p* < 0.05). These differences suggest that the observed effect is not solely caused by the nanoparticles but rather by their drug content. Comparison of the results for apoptosis in the two studied lines, C6 and V79-4, revealed a higher percentage of apoptotic cells in the C6 tumor line, particularly in samples containing encapsulated drugs (MTG2, MTCG2, NTCG2). The differences shown in Figure 8 are statistically significant (*p* < 0.05) and suggest an increased sensitivity of tumor cells to the nanoparticle with co-encapsulated TMZ and curcumin treatment. This trend may indicate the selectivity of the tested HSA-based nanoparticles in therapy, which is an essential advantage in cancer treatment.

Several studies have shown that the free form of some chemotherapeutics (e.g., doxorubicin, paclitaxel, TMZ) can induce a higher level of apoptosis compared to the form encapsulated in albumin nanoparticles when both are administered at the same concentration of formulation (not active drug), particularly in short-term in vitro experiments (24–48 h). For example, free doxorubicin induced significantly higher apoptosis than the variant encapsulated in magnetic nanoparticles at the same total administered dose due to the immediate availability of the free form [99]. Similarly, nab-paclitaxel did not overcome the effect of free paclitaxel on A549 cells at 24 h due to slower uptake by endocytosis [100,101]. In the case of TMZ, most nanoformulations are designed to improve brain delivery and stability. However, in vitro studies show that sustained release of TMZ may have a slower immediate effect than free TMZ. For example, free TMZ dissolves and takes effect quickly, usually within a few hours, whereas TMZ encapsulated in polymer or albumin nanoparticles is released gradually over a period of tens of hours [102]. At the same concentration of nanoparticles, the initial amount of active TMZ available is significantly lower than that of the free dosage, resulting in a reduced initial rate of apoptosis. Similarly, curcumin, a compound known for its low bioavailability, benefits from various nanoformulations that gradually increase cellular exposure to the active agent. Research indicates that albumin nanoparticles encapsulating curcumin release it slowly, allowing effective concentrations to be sustained over a more extended period.

In contrast, the free form of curcumin (when properly dissolved) can quickly induce cytotoxicity and apoptosis before the nanoparticles have released their entire load of the active substance [102]. This difference is primarily due to the slow release of the drug from the nanoparticles, delayed cellular uptake via endocytosis mechanisms, and limited access of the active content to the cytoplasm during the first hours post-treatment. In contrast, the free form of the drug is readily available and diffuses quickly into cells, reaching effective intracellular concentrations that can prompt the activation of apoptotic pathways. Therefore, without a long-term kinetic evaluation or a dose–response study, it is possible that nanoparticles do not reflect their full therapeutic potential in vitro, thereby explaining the reduced apoptotic response observed compared to free drug treatment.

#### 2.4.2. Utilization of in Vitro Blood–Brain Barrier Models and Assessment of the Permeability of Functionalized Nanoparticles Containing Co-Encapsulated Drugs

**Figure 9:**
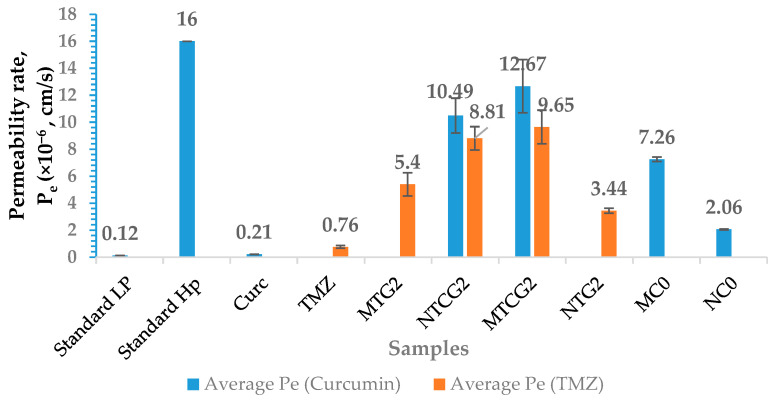
Permeability rate P_e_ for the analyzed samples (*p* < 0.05).

The method for testing the permeability of nanoparticles through the BBB is described in the Section 4 below. The in vitro parallel artificial permeability assay (PAMPA, Kit PMBBB-096 from BioAssay Systems (Hayward, CA, USA)) was employed to evaluate whether nanoparticles can cross the BBB. The results obtained for determining the permeability rate are presented in Figure 9 for MTG2, MTG2, NTCG2, MTCG2, NTG2, MC0, NC0, and free active principles (curcumin and TMZ). As a negative standard, we used the low-permeability (LP) and high-permeability (HP) standards from the PAMPA-BBB kit from Bioassay.

Verapamil was used as a high-permeability standard (P_e_ = 16 × 10^−6^ cm/s), and theophylline was used as a low-permeability standard (P_e_ = 0.12 × 10^−6^ cm/s) [103].

Research indicates that free drugs generally cannot cross the BBB, with permeability (P_e_) values extremely low at 0.21 × 10^−6^ cm/s for curcumin and 0.75 × 10^−6^ cm/s for TMZ. Although TMZ has proven to be clinically effective in treating brain tumors and has shown the ability to overcome the blood–brain barrier (BBB) in vivo, the PAMPA-BBB assay indicated a permeability value (P_e_) of 0.78 × 10^−6^ cm/s, categorizing it as having low permeability. This discrepancy can be attributed to the limitations of the PAMPA model, which assesses only passive diffusion and does not take into account active transport or other physiological mechanisms that occur in vivo. In contrast, nanoparticles demonstrate considerably higher Pe values, regardless of the specific type examined. The only drugs that can effectively cross the BBB and have a high permeability are those co-encapsulated in nanoparticles based on HSA that are functionalized or not with LMWP, specifically, MTCG2. These nanoparticles exceed the established P_e_ value of 10 × 10^−6^ cm/s, as mentioned in the literature [104]. Considering that free curcumin has the lowest P_e_ value and that the P_e_ value of curcumin in the aforementioned nanoparticles is higher than that of the standard, we can conclude that these nanoparticles can overcome the BBB. We conducted the PAMPA-BBB permeability test on nanoparticles containing temozolomide, specifically, modified with LMWP (such as MTG2) and unmodified NTG2. Our results show that both types of nanoparticles with TMZ encapsulated exhibit an average permeability. However, the permeability for MTG2 is slightly higher, although the difference between the two samples is not statistically significant (*p* > 0.05). The test was also performed on drugs encapsulated in non-crosslinked nanoparticles with GO, which were either functionalized with LMWP (MC0) or not (NC0), and contained encapsulated curcumin.

The results showed that the non-functionalized NC0 nanoparticles have a low capacity to cross the BBB, with a permeability value of 2.06 × 10^−6^ cm/s, while the MC0 functionalized nanoparticles show a higher average permeability of 7.26 × 10^−6^ cm/s. The differences between these samples are statistically significant, with a *p*-value of 0.02. These findings demonstrate the role of the LMWP peptide in enhancing passive permeability through the blood–brain barrier, especially in nanoparticle-based drug delivery systems. We note that the permeability rate P_e_ of the MTG2 sample increases seven times compared to free TMZ (*p* < 0.05), indicating that LMWP-functionalized nanoparticles can enhance permeability across the BBB.

The synergistic effect of curcumin and TMZ is evident in both unmodified NCTG2 nanoparticles and MTCG2-modified nanoparticles, where the Pe value increases compared to that of free drugs or LMWT-modified nanoparticles containing TMZ. However, the P_e_ value for NCTG2 is lower than that of functionalized nanoparticles but higher compared with that for TMZ from MTG2 or NTG2 samples, or for curcumin encapsulated in MC0 or NC0 samples, indicating a synergistic effect between TMZ and curcumin (*p* < 0.05). The particle types that can cross the BBB with a high permeability value are MTCG2 and NCTG2, which are determined by the synergistic effect between curcumin and TMZ, enhancing the hydrophobicity of the nanoparticles (they contain a higher amount of drug). The permeability of the nanoparticles through the BBB also depends on the functionalization of albumin nanoparticles with LMWP. Similar results were obtained for meloxicam-loaded albumin particles, with a P_e_ value of approximately 12.5 × 10^−6^ cm/s. The researchers explained this effect as being due to the characteristics of the nanoparticles and the drug release kinetics [105].

#### 2.4.3. Evaluation of Antioxidant Activity

**Figure 10:**
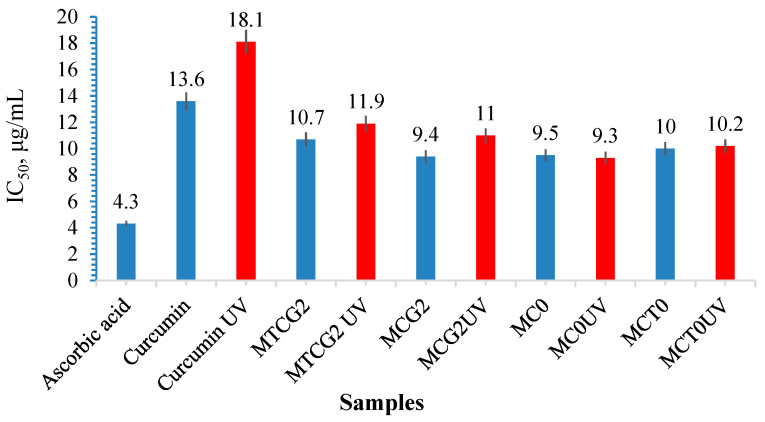
IC_50_ values obtained after testing antioxidant activity using the DPPH assay for ascorbic acid, free curcumin, UVA-irradiated free curcumin, and curcumin encapsulated in HSA nanoparticles obtained through the self-assembly method, whether irradiated or non-irradiated with UV.

The antioxidant activity was assessed for free curcumin, UV-irradiated curcumin, ascorbic acid, and curcumin incorporated in HSA nanoparticles crosslinked with aldehyde groups derived from OG obtained through self-assembly. The results are illustrated in Figure 10. To evaluate whether the nanoparticles provide a protective effect for curcumin and if they can be sterilized with UV light at a wavelength of 365 nm, a portion of the nanoparticles containing immobilized curcumin was exposed to a UV light source for 30 min.

The antioxidant activity can be diminished if curcumin degrades. As the IC_50_ value increases, the antioxidant activity of the samples decreases. Figure 10 shows that the IC_50_ values for ascorbic acid, free curcumin, and curcumin encapsulated in nanoparticles are close to or lower than that of free curcumin. For free curcumin exposed to UVA light, we note that the IC_50_ value increases compared to free curcumin that has not been exposed to UVA light. For curcumin extracted from samples, whether irradiated or not with UVA, we find that the antioxidant activity increases compared to that of free curcumin not exposed to UV (i.e., the IC_50_ decreases). The increased antioxidant activity of curcumin encapsulated in HSA nanoparticles may be attributed to albumin. For curcumin from self-assembled nanoparticles irradiated by UV, we observe that the IC_50_ value is slightly higher compared to the same samples that were not irradiated, but it does not exceed the IC_50_ value of free curcumin, leading us to conclude that immobilization allows the polyphenol to retain its antioxidant activity, while the polymer matrix provides protective support for curcumin against factors that may cause its degradation.

For samples with encapsulated polyphenol irradiated with UV light at 365 nm, it is observed that curcumin retains its antioxidant activity, showing an IC_50_ value lower than that of free curcumin or UV-irradiated free curcumin, even though the IC_50_ value is slightly higher compared to curcumin that was not UV-irradiated (Figure 10). Curcumin maintains its antioxidant activity, and the polymer matrix serves as a protective barrier against factors that can lead to its degradation. The enhanced antioxidant activity of curcumin encapsulated in HSA-based nanoparticles may be attributed to albumin’s own antioxidant properties, as demonstrated in a previous study [46]. Research has shown that BSA-dextran nanoparticles with immobilized curcumin can enhance the cellular antioxidant activity of curcumin in Caco-2 cells [106]. A study demonstrated that curcumin can interact with ovalbumin, altering the protein’s secondary structure and making it more susceptible to heat-induced decomposition.

Additionally, it was confirmed that the resulting compound exhibited increased solubility and improved antioxidant properties compared to pure curcumin [107]. In a study, sodium alginate composite particles containing ZnO with encapsulated curcumin were exposed to UV light for 6 h. The results showed that the DPPH free radical scavenging capacity decreased by 13.7% for encapsulated curcumin compared to free curcumin, for which the DPPH free radical scavenging capacity decreased by 62.04% [108]. Some research indicates that curcumin generates singlet oxygen and other reactive oxygen species upon exposure to UV light or photodegradation [109].

#### 2.4.4. Evaluation of the Cytotoxicity of Drug-Encapsulated Particles via the MTT Assay

**Figure 11:**
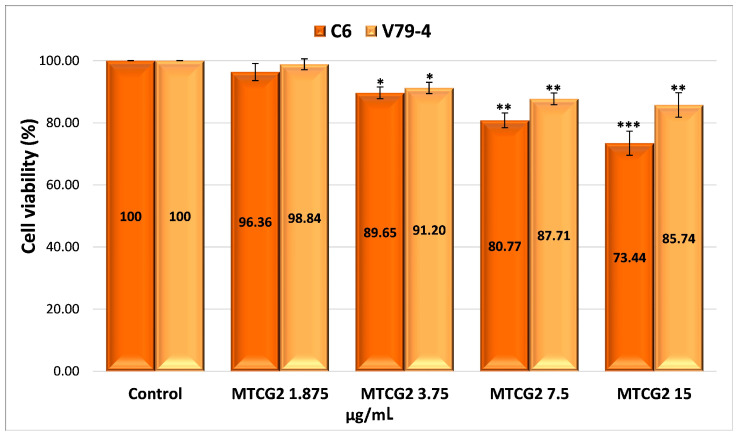
The viability of C6 tumor cells and normal V79-4 cells after treatment with MTCG2 at various doses for 48 h (* *p* < 0.05, ** *p* < 0.01, *** *p* < 0.001).

In the first step, self-assembled nanoparticles that were crosslinked with OG, with or without encapsulation of TMZ and curcumin, were tested. Figure 11 shows the viability of C6 tumor cells and normal V79-4 cells after treatment with self-assembled HSA nanoparticles MTCG2, MTG2, NTCG2, MCG2, curcumin, and TMZ at different doses for 48 h. The MCG2 sample served as a control for the samples with encapsulated drugs, and untreated cells were used as a negative control for MG2.

**Figure 12:**
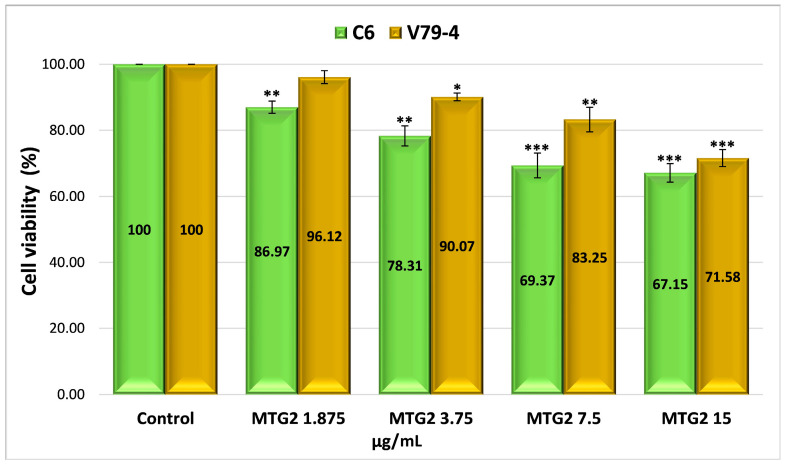
Viability of C6 tumor cells and normal V79-4 cells after treatment with MTG2 at different doses for 48 h (* *p* < 0.05, ** *p* < 0.01, *** *p* < 0.001).

In the first step, nanoparticles obtained through self-assembly that were crosslinked with OG and either co-encapsulated or not with TMZ and curcumin were then tested. After applying the treatment with the MTCG2 sample, the viability of normal and tumor cells did not change significantly compared to the control. Thus, the percentage of live cells for the C6 tumor line varied between 96.36% (at the minimum concentration analyzed, 1.875 μg/mL) and 73.44% at the maximum concentration of 15 μg/mL. For the normal cell line V79-4, the percentage of cell viability ranged from 98.84% (1.875 μg/mL) to 85.74% (15 μg/mL), indicating a lower cytotoxic effect compared to the tumor line, as illustrated in Figure 11. Treatment with MTG2 nanoparticles obtained from LMWP-modified albumin, which were crosslinked with OG and contained only encapsulated TMZ, demonstrated lower cell viability percentages in both tested cell lines compared to nanoparticles that included both encapsulated drugs, curcumin and TMZ. Specifically, the C6 tumor cell line exhibited a live cell percentage ranging from 86.97% at a concentration of 1.875 μg/mL to 67.15% at the maximum concentration of 15 μg/mL. The latter figure indicates a significant cytotoxic effect of 32.85%. In contrast, the normal V79-4 cell line showed a lower cytotoxic effect than the tumor cell line, with live cell percentages ranging from 96.12% at 1.875 μg/mL to 71.58% at 15 μg/mL, as illustrated in Figure 12.

**Figure 13:**
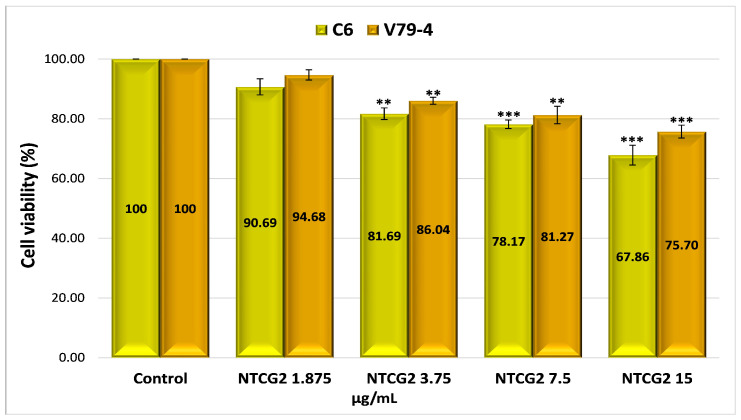
Viability of C6 tumor cells and normal V79-4 cells after treatment with NTCG2 at different doses for 48 h (** *p* < 0.01, *** *p* < 0.001).

Albumin nanoparticles crosslinked with OG, which co-encapsulate TMZ and curcumin and are designated as NTCG2, generally produced lower cell viability values compared to the modified MTCG2 nanoparticle variant. In the case of the C6 tumor cell line, cell viability ranged from 90.69% at 1.875 μg/mL to 67.86% at 15 μg/mL. Normal V79-4 cells showed a viability of 94.68% at 1.875 μg/mL and 75.70% at 15 μg/mL, as illustrated in Figure 13.

**Figure 14:**
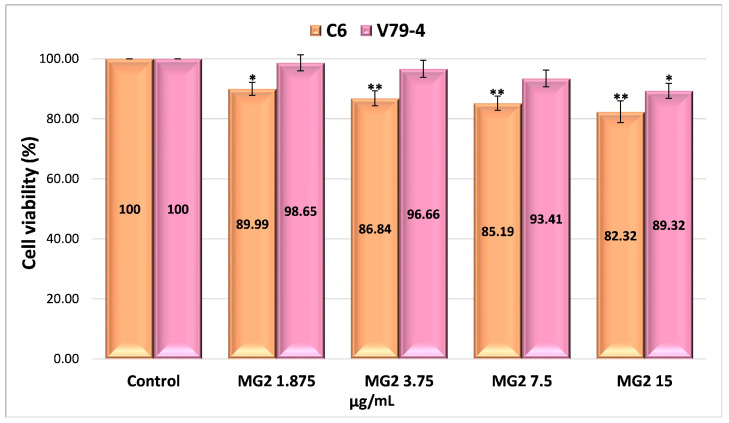
Viability of C6 tumor cells and normal V79-4 cells following treatment with MG2 at various doses over 48 h (* *p* < 0.05, ** *p* < 0.01).

The effects of modified albumin nanoparticles, which were not loaded with drugs, were examined on both cell lines. The albumin nanoparticles, modified with LMWP and crosslinked with OG, did not induce significant changes in cell viability compared to the control. Specifically, the percentage of viable cells for the neoplastic C6 cell line ranged from 89.99% at a minimum concentration of 1.875 μg/mL to 82.32% at a maximum concentration of 15 μg/mL. For the normal V79-4 cell line, cell viability ranged from 98.65% (at 1.875 μg/mL) to 89.32% (at 15 μg/mL), indicating lower cytotoxic effects compared to the tumor line. As shown in Figure 14, the percentage of dead cells at 15 μg/mL was 10.68%, confirming the non-toxic nature of the nanoparticles, as per ISO 10993-5 [110].

**Figure 15:**
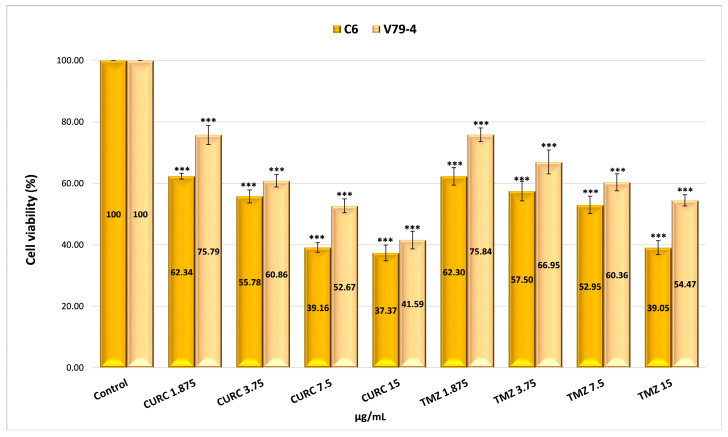
Viability of C6 tumor cells and normal V79-4 cells after treatment with CURC and TMZ at different doses for 48 h (*** *p* < 0.001).

In the experiments involving tumor and normal cell cultures, free drugs were tested, demonstrating a strong cytotoxic effect with varying intensities on both cell lines. After 48 h of treatment with curcumin (CURC) at different doses, the viability of normal and tumor cells changed significantly compared to the untreated control. For the C6 tumor line, the percentage of viable cells ranged from 62.34% at the minimum concentration tested (1.875 μg/mL) to 37.37% at the maximum concentration (15 μg/mL), indicating remarkable cytotoxic effects of 37.66% and 62.63%, respectively. In the case of the normal cell line V79-4, the percentages of cell viability ranged from 75.79% at 1.875 μg/mL to 41.59% at 15 μg/mL. Although the cytotoxic effects were lower compared to the tumor line, they were still significant, with a cytotoxic action of 58.41%, as shown in Figure 15. Treatment with TMZ, administered at the same doses as curcumin, produced significant cytotoxic effects on both cell lines, with a more pronounced impact on the tumor cells. After 48 h of treatment, the viability of C6 glioma cells ranged from 62.30% at the minimum dose to 39.05% at the maximum dose, indicating cytotoxic effects of 37.70% and 60.95%, respectively. A similar, albeit less intense, effect on cell viability was observed in the normal V79-4 cells, which varied between 75.84% (1.875 µg/mL) and 54.47% (15 µg/mL), as illustrated in Figure 15.

To evaluate the results, the IC_50_ value was determined based on the concentration of the drug incorporated in the nanoparticles.

Additionally, the concentration of the encapsulated drug was significantly lower than that of the free drug. The IC_50_ value was calculated based on the drug concentration in the nanoparticles to facilitate comparison of the results. This IC_50_ value was derived from a graph plotting the percentage of dead cells against the drug concentration in the samples. Table 6 displays the results obtained from the study. A lower IC_50_ value indicates the higher cytotoxicity of the samples. From this table, it is evident that encapsulated drugs exhibit a more pronounced cytotoxic effect compared to free drugs.

Treatment of C6 glioma tumor cells and normal V79-4 cells with TMZ resulted in significant cytotoxic effects on both cell lines, with a more substantial impact observed in the tumor cells. After 48 h of exposure to TMZ, the viability of the neoplastic C6 cells ranged from 65.12% at the lowest dose to 40.82% at the highest dose. These values corresponded to marked cytotoxic effects of 34.88% and 59.18%, respectively. In contrast, the normal V79-4 cells exhibited a similar, though less pronounced, decrease in viability, ranging from 76.54% at a dose of 1.875 µg/mL to 55.19% at the maximum dose of 15 µg/mL. Additionally, it is essential to note that both drugs exhibit low solubility in culture medium or phosphate buffer [111]. The cytotoxicity of 0.1% DMSO was evaluated for both sets of tests. The results obtained highlight its non-toxic nature, with cell viability comparable to that of the control sample, specifically, 98.64% (for the C6 tumor line) and 99.85% (for the V79-4 cell line). These results align with recent studies [112,113,114] that discuss the cytotoxic potential of nanoparticles loaded with curcumin or TMZ, with or without other natural or synthetic compounds, to optimize targeted therapy in tumor diseases.

The results of the cytotoxicity evaluation indicated that albumin nanoparticles, obtained through self-assembly and crosslinked with OG, exhibited varying levels of cytotoxic effects. These effects depended on the type of nanoparticle tested, the concentration used, and the specific cell line studied. Notably, the cytotoxic effect for the C6 tumor cell line was significantly stronger than that observed in normal V79-4 cells. This observation demonstrates a selective response, which is beneficial for utilizing these vectors in anticancer therapy. Additionally, the functionalized nanoparticles showed a more pronounced cytotoxic effect than the unfunctionalized ones. According to the literature reviewed, the drug-free nanoparticles (MG2) are classified as non-toxic compounds.

In contrast, other compounds show a weak cytotoxic effect, with cell viability values ranging from 60% to 80%. Cells exposed to curcumin and TMZ exhibited viability below 40%, indicating a strong cytotoxic response. This response is likely due to the low solubility of these drugs, and the results are influenced by the dosage used.

The results from cytotoxicity testing correlated with the encapsulated drug data (Table S3 and Table 6), highlighting an essential aspect of the tested systems’ efficiency.

The higher IC_50_ value observed for free drugs may be attributed to their low solubility.

The MTCG2 sample contained a lower total concentration of encapsulated drug compared to the NTCG2 sample, with values of 0.78 µg/mL and 1.048 µg/mL, respectively, at a particle concentration of 7.5 µg/mL. Despite this difference, the viability of C6 tumor cells was almost equal at this concentration for both samples. Additionally, the IC_50_ values for MTCG2 and NTCG2 were 2.93 µg/mL and 3.62 µg/mL, respectively. These results indicate that MTCG2 was more efficient in treating the C6 cell line at lower drug concentrations. The results are further supported by a comparison with the MTG2 sample, which contains only TMZ and is functionalized. Although the concentration of TMZ encapsulated in MTG2 is low (0.148 µg/mL at a concentration of 7.5 µg/mL nanoparticles), it produced the lowest IC_50_ value on the C6 cell line (0.51 µg/mL), highlighting the role of nanoparticle functionalization with LMWP in enhancing the therapeutic effect of the drug at low doses. The increased effectiveness is likely due to the nanoparticles being internalized into the cells, and this is especially notable since the size of the MTG2 nanoparticles is smaller than that of the other two samples.

Additionally, functionalization with LMWP may further improve the internalization of these nanoparticles into the cells. The NTCG2 sample contains higher concentrations of TMZ and curcumin, specifically, 0.64 µg/mL of curcumin and 0.408 µg/mL of TMZ, when combined with 7.5 µg/mL of nanoparticles. However, because it is not functionalized with LMWP, it exhibits a lower cytotoxic effect, as indicated by a higher IC_50_ value, demonstrating that both the quantity of the drug and the effectiveness of the delivery system significantly impact therapeutic outcomes. These observations align with the existing literature, which shows that LMWP enhances the cellular uptake of nanoparticles, promotes diffusion into tumor tissue, and facilitates intracellular drug release. This results in improved treatment efficacy at lower concentrations of the active substance encapsulated in functionalized nanoparticles [57,115].

#### 2.4.5. Analysis of the Hemocompatibility of Nanoparticles

**Figure 16:**
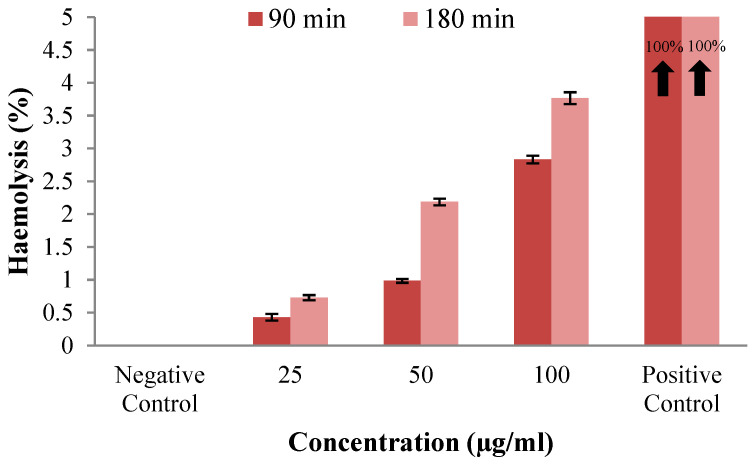
Degree of hemolysis of LMWP-modified HSA nanoparticles without encapsulated drugs, produced via self-assembly and crosslinked with OG, namely, MG2.

For the hemolysis test, the MG2 sample without encapsulated drugs was chosen. The results obtained are shown in Figure 16:

The information obtained from this test suggests that all LMWP-modified HSA nanoparticles demonstrate good compatibility with the blood environment, remaining within normal limits (<10% compared to the positive control sample), even at high concentrations of the suspensions used [58,116].

HSA-based nanoparticles have generally demonstrated hemocompatibility, exhibiting a low risk of hemolysis, with values below 5% in most studies. For instance, this behavior was highlighted in the case of HSA–arsenite trioxide nanoparticles [117] and in the case of unfunctionalized HSA nanoparticles, which induced minimal or even negligible hemolysis [118,119]. Additionally, it has been observed that surface modifications involving cationic charge can mildly increase the hemolytic potential; however, careful engineering of these characteristics allows for the maintenance of hemocompatibility within safe limits [120].

### 2.5. Stability Evaluation

#### 2.5.1. Study of the pH Stability of Drugs Encapsulated in Nanoparticles

Curcumin undergoes chemical degradation in aqueous solutions, and this degradation intensifies with increasing pH values. Even at physiological pH, curcumin is rapidly degraded into bicyclopentadione, vanillin, and ferulic acid [109]. It is essential to determine whether encapsulating curcumin in albumin-based particles can prevent its degradation in solutions with varying pH levels. The results obtained for the analyzed samples are presented in Table 7. Research indicates that HSA-based nanoparticles, whether modified with LMWP or not, effectively protect curcumin from degradation in buffer solutions with varying pH levels. The half-life (t_1/2_) of curcumin encapsulated in nanoparticles is significantly longer than that of free curcumin, regardless of the pH. For HSA nanoparticles created through self-assembly, specifically, MTCG2 and NTCG2, the t_1/2_ values of encapsulated curcumin are higher at pH levels of 6.8 and 7.4 compared to the t_1/2_ values observed at pH levels of 3 and 9.

The degradation of curcumin in buffer solutions with varying pH levels occurs spontaneously due to the presence of oxygen-free radicals [121]. pH stability tests were conducted on curcumin encapsulated in HSA nanoparticles. The findings revealed that HSA particles offer protection to curcumin, resulting in a slower degradation rate of the encapsulated curcumin in both acidic and basic pH environments when compared to free curcumin (Table 7). At pH 3, albumin may degrade via acid hydrolysis, weakening the polymer matrix and resulting in a shorter degradation half-life compared to the half-life at pH 6.8 or 7.4. At pH 9, the OG used for crosslinking the albumin-based nanoparticles has free carboxylate groups that can cause electrostatic repulsions among macromolecules. This phenomenon facilitates the relaxation of the polymer network, allowing for increased solution absorption within the particles at pH 9, which might lead to curcumin degradation. All samples analyzed showed a half-life greater than 19 h, regardless of the pH value, indicating that whether HSA nanoparticles are modified with LMWP through self-assembly or are unmodified, they effectively protect curcumin from environmental effects.

Additionally, the half-life for degradation is higher for curcumin incorporated into nanoparticles based on modified albumin compared to that for curcumin in nanoparticles derived from unmodified HSA. This difference is likely due to the amine groups of LMWP, which can interact more effectively with the functional groups in OG, thereby enhancing the stability of the nanoparticles. Alternatively, intermolecular interactions, such as hydrogen bonds, may form between the macromolecules and the drug, thereby reducing the diffusion of the buffer solution at different pH levels within the particles. Moreover, statistically significant differences exist between the percentage of degradation of free curcumin at varying pH levels and that of curcumin included in nanoparticles, with a *p*-value of less than 0.05. HSA-based nanoparticles are a promising delivery system that extends the systemic circulation time of active compounds, particularly those with low bioavailability. In the literature, the half-lives of various active substances encapsulated in albumin nanoparticles are relatively short. For instance, doxorubicin encapsulated in HSA-based nanoparticles exhibited a half-life of 19.1 ± 1.7 h when administered intravenously [122]. The clinically approved formulation, Abraxane^®^ (which contains paclitaxel in HSA), also demonstrated a half-life ranging from 20.61 to 27.31 h based on clinical studies involving patients [123].

In this study, we found that the degradation half-life of curcumin encapsulated in low-molecular-weight protamine (LMWP)-modified HSA nanoparticles, crosslinked with OG, is up to 53 h at pH 7.4. This duration significantly surpasses all previously reported values for similar systems. The extended degradation half-life can be attributed to the combined effect of the internal network stabilized by OG and the molecular protection offered by LMWP functionalization. The developed administration system exhibits effective encapsulation abilities and enhanced stability against degradation across various pH levels, making it a promising option for systemic applications targeting the central nervous system.

#### 2.5.2. Study of the Light Stability of Drugs Encapsulated in Nanoparticles

Assuming that the polymer matrix can protect curcumin from photodegradation, a comparative study was conducted on free curcumin, as well as on HSA-based nanoparticles that were not crosslinked with OG (samples NC0, MC0, MTC0, and NTC0), and on crosslinked samples with OG (samples MTCG2, NTCG2, and MTCG3). When free curcumin was exposed to natural light and air, it was observed that the absorbance values at 425 nm decreased over time. The percentages of curcumin that degraded over time in air and light, as analyzed from the samples, are presented in Table 8.

Research indicates that all HSA-based nanoparticles effectively protect curcumin from degradation caused by exposure to light and air. Free curcumin showed a degradation rate of 32.3% after 30 days under these conditions. Among the self-assembled HSA nanoparticles, the modified versions—particularly MTCG2—offer better protection for curcumin than the unmodified HSA nanoparticles, such as NTCG2. Notably, both types of nanoparticles co-encapsulated curcumin and TMZ. It is well known that curcumin is light-sensitive, being degraded (discolored) when exposed to natural light. The results showed that curcumin is better protected in the polymer matrix than in the unimmobilized form. It was observed that the degree of crosslinking of the nanoparticles affects the stability of encapsulated curcumin; nanoparticles with a higher degree of crosslinking, specifically, MCTG3 (with a molar ratio between amine and aldehyde groups of 1:1), provide superior protection to curcumin compared to MCTG2 nanoparticles, which have a molar ratio of 1:0.75. A higher degree of curcumin degradation is observed in the uncrosslinked MCT0 samples, with a recorded degradation of 19.8%, supporting the previous hypothesis regarding the degree of crosslinking. For the LMWP-functionalized HSA-based nanoparticles that exclusively encapsulated curcumin, such as MC0, a degradation rate of only 10.7% is noted compared to the MCT0 sample (which co-encapsulated both curcumin and TMZ), indicating that TMZ may induce structural changes in the immobilized curcumin, leading to a higher degradation percentage over time. There are statistically significant differences in the degradation percentage between free curcumin and curcumin incorporated in nanoparticles, *p* < 0.0.

#### 2.5.3. Thermogravimetric Analysis

**Figure 17:**
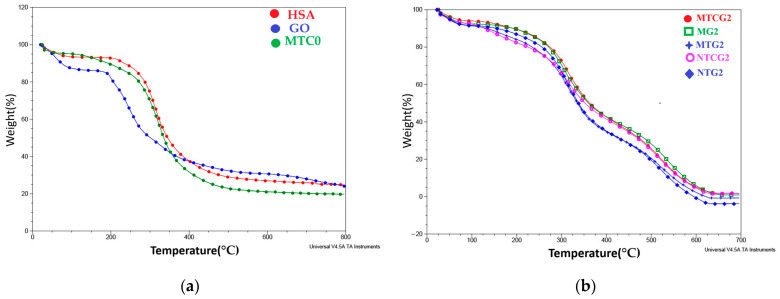
Thermogravimetric curves for (**a**) HSA, OG, and sample MCT0 (not crosslinked with OG) and (**b**) samples that are crosslinked with GO, both with and without encapsulated drugs (MG2, MTG2, NTCG2, NTG2, MTCG2).

Figure 17 presents the thermogravimetric (TGA) curves for HSA, OG, and the nanoparticles obtained, both with and without the co-encapsulated active principles. Analyzing these curves provides valuable insights into the thermal stability of each component and the effects of encapsulation on the thermal behavior of the delivery system.

In the thermogravimetric analysis of HSA (Figure 17a), two distinct weight loss stages are observed. The first takes place between 20 °C and 267.19 °C, resulting in about 15% weight loss, which is due to the evaporation of a small amount of moisture. The second stage begins at approximately 267.19 °C and continues to 403.39 °C, with a significant weight loss of 62.77%, corresponding to the decomposition of protein. The residual weight of HSA after these processes is 25.79%. In the case of OG (see Figure 17a), the initial stage of the thermal process begins at 20 °C and concludes at 170 °C, resulting in approximately 15% weight loss. The second stage, which involves decomposition, starts at 170 °C and continues until approximately 299 °C, resulting in a 53.6% weight loss. After this, the amount of OG decreases slightly, leaving a residue of 29.87%.

For the LMWP-modified HSA nanoparticles labeled MCT0 (see Figure 17a), which are not crosslinked with OG and contain both co-encapsulated drugs, the process of water evaporation begins at 25 °C and continues until 288.27 °C. This phase results in a 20.62% weight loss. The subsequent phase, which involves thermal decomposition, starts at 288.27 °C and ends at 422.43 °C, resulting in an additional weight loss of 50.8%. Ultimately, the remaining residue constitutes 20.21% of the initial weight. In the study of LMWP-modified albumin nanoparticles crosslinked with OG and not containing incorporated drugs, several degradation stages were observed for sample MG2 (Figure 17b). The first stage involves moisture evaporation, which occurs between 25 °C and 283.27 °C, resulting in a weight loss of 23.44%. The second stage of thermal decomposition begins at 283.27 °C and continues until 499.75 °C, leading to an additional weight loss of 48.32%. A third stage, which appears only in the crosslinked samples with OG, is due to the oxidation of carbon residues. This stage starts at 499.75 °C and ends at 605.58 °C, resulting in a weight loss of 23.21%. Ultimately, the remaining residue after these processes is 0.96%. For LMWP-modified albumin nanoparticles, crosslinked with OG and encapsulating TMZ, MTG2 (Figure 17b), it is observed that the first stage of moisture evaporation, which is observed from 25·°C to 286.74·°C, results in a weight loss of 30.98% in this stage. The second stage, the thermal decomposition stage, occurs between 286.74 °C and 474.45 °C, corresponding to a weight loss of 44.02%. The third stage begins at 474.45 °C and continues to 598.12 °C, resulting in a weight loss of 22.43%. The amount of residue was 0.63%.

For LMWP-modified albumin nanoparticles, crosslinked with OG containing co-encapsulated TMZ and curcumin, MTCG2 (Figure 17b), it is observed that water evaporation begins at 25 °C and continues up to 279.1 °C, resulting in a weight loss of 21.16%. The second stage, involving thermal decomposition, takes place between 279.1 °C and 462.68 °C, with a corresponding weight loss of 45.82%. The third stage begins at 462.68 °C and extends to 589.78 °C, with a weight loss of 26.65%. The remaining residue amounts to 2.36%. For unmodified albumin-based nanoparticles with LMWP, crosslinked with OG and containing TMZ encapsulated as NTG2, it is observed that the initial water evaporation begins at 25 °C and continues until 251.44 °C, resulting in a weight loss of 19.21%. The second stage, involving thermal decomposition, begins at 251.44 °C and extends to 432.67 °C, resulting in a weight loss of 50.06%. The third stage starts at 432.67 °C and reaches 556.24 °C, resulting in a weight loss of 24.21%. The remaining residue accounts for 2.38%. For unmodified albumin nanoparticles crosslinked with OG, co-encapsulating TMZ and curcumin (NTCG2), it is observed that the first stage of water evaporation occurs from 25 °C to 239.67 °C, with a corresponding weight loss of 21.67%. The second stage, involving thermal decomposition, was observed between 239.67 °C and 407.37 °C, with a corresponding weight loss of 37.87%. The third stage begins at 407.37 °C and increases to 525.64 °C, resulting in a weight loss of 21.85%. The remaining residue was 2.1%.

Thermogravimetric analysis revealed that all nanoparticle samples exhibit higher temperatures at the start of degradation and peak degradation temperatures within elevated ranges, along with significantly lower final residues compared to their precursors (HSA and GO). Specifically, the crosslinking of amino groups in albumin with aldehyde groups from OG leads to the appearance of a third degradation stage (carbonic oxidation with a maximum temperature exceeding 500 °C), indicating improved thermal stability and complete decomposition of organic components. The modification with LMWP and the encapsulation of TMZ or curcumin do not negatively impact the stability of the polymer matrix. Instead, these processes introduce a complex moisture evaporation step, promoting complete degradation and resulting in residuals of less than 3%. Additionally, samples with encapsulated drugs undergo a gradual, multi-stage degradation, which reflects the contribution of each component to the overall thermal behavior of the nanoparticles. Although these samples showed a lower final residue amount, the high temperatures at which the decompositions were observed suggest that the resulting nanoparticulate delivery systems are more thermally stable than the free precursors. These findings indicate that the preparation method and the crosslinking with OG significantly affect the thermal behavior of the nanoparticles, which is a critical factor for their stability during processing and storage. In conclusion, the improved thermal stability may support the use of these systems in stable pharmaceutical formulations.

The stability of the nanoparticles was evaluated through thermogravimetric analysis (TGA) and stability experiments of both free and encapsulated curcumin under various conditions, including pH, light exposure, and air exposure. These assessments offer valuable insights into the physicochemical properties of the system under conditions that simulate physiological or storage stress. However, we recognize that we did not perform serum stability tests and protein corona analysis, which represents a limitation of this research. We intend to address this in future studies.

## 3. Conclusions

The results indicate that HSA-based nanoparticles can be functionalized with LMWP to enhance their intracellular diffusion and ability to overcome the BBB. These nanoparticles were obtained using the self-assembly method and were crosslinked with OG, using different molar ratios between the NH_2_ groups of HSA and the CHO groups of GO. Most nanoparticles have sizes below 150 nm, which indicates their potential for effective diffusion through the BBB. Two active principles, TMZ and curcumin, were co-encapsulated in the HSA-based nanoparticles. Curcumin showed a higher immobilization efficiency due to stronger hydrophobic interactions with HSA.

The in vitro release kinetics study indicated TMZ exhibited higher release efficiency (RE%) values from nanoparticles at pH 4, while curcumin showed a greater RE% value at pH 7.4. Decreasing the crosslinking degree increased the RE% for both active compounds. Cytotoxicity tests on C6 tumor cells and normal V79-4 cells showed a significant antitumor effect, which was associated with the induction of apoptosis. The cytotoxicity induced by HSA-based nanoparticles varied depending on several factors, including the specific nanoparticle formulation, the concentration used for assessment, the dosage of the encapsulated drug, and the type of cell line involved. This effect was especially pronounced in tumor cells, highlighting a valuable selectivity for anticancer therapy. The permeability evaluation using the PAMPA-BBB membrane diffusion assay demonstrated that functionalized HSA-based nanoparticles have higher BBB permeability compared to free active compounds or non-functionalized nanoparticles. HSA-based nanoparticles that co-encapsulate both TMZ and curcumin, especially those functionalized with LMWP, demonstrated greater permeability through the PAMPA-BBB membrane compared to other functionalized nanoparticles containing only one of the drugs. The in vitro results indicate a potential for the drugs to cross the BBB and suggest a possible synergistic effect between them that could be beneficial in overcoming the BBB. However, these observations need to be validated through in vivo studies for confirmation. The role of functionalized LMWP on HSA-based nanoparticles in facilitating the transport of nanoparticles across the BBB is supported by the experimental data presented, but further research is necessary to confirm this mechanism. The antioxidant activity of curcumin was assessed using the DPPH assay. Under UV light, encapsulated curcumin demonstrated superior antioxidant activity compared to the free form due to the protective role of the polymer matrix for curcumin. Encapsulating curcumin in nanoparticles protects it from degradation factors such as light and pH, significantly increasing its degradation half-life (at a pH of 9, the t_1/2_ of encapsulated curcumin is 21 h, whereas free curcumin has a t_1/2_ value of only 2.3 h). These results demonstrate the therapeutic potential of HSA-based nanoparticles containing co-encapsulated TMZ and curcumin for treating brain diseases, particularly cancer. However, additional in vivo studies are necessary to confirm the efficacy of these systems.

## 4. Materials and Methods

### 4.1. Materials

The deacetylated gellan used to obtain the hydrogel particles was purchased from Kelkogel (London, UK) and has a molecular weight between 2 × 10^5^ and 3 × 10^5^ Da. It was oxidized in the presence of NaIO4 [52] to yield oxidized gellan. Human serum albumin (lyophilized powder), protamine (amorphous powder), thermolysin from Geobacillus stearothermophilus, ninhydrin, Tween 80, polyethylene glycol sorbitan monooleate, 2,2-diphenyl-1-picrylhydrazyl (DPPH), EDTA, and disodium salt of ethylenediaminetetraacetic acid dihydrate were purchased from Sigma Aldrich (Darmstadt, Germany). SMCC, succinimidyl-trans-4-(N-maleimidomethyl)cyclohexane-1-carboxylate, was purchased from MerckMillipore (Darmstadt, Germany). Disodium phosphate (Na_2_HPO_4_·12H_2_O), monosodium phosphate (NaH_2_PO_4_·2H_2_O), and sodium acetate were obtained from Chemical Company. Solvents: Dimethyl sulfoxide (DMSO) and methanol were purchased from Sigma Aldrich. Ethyl alcohol (C_2_H_5_-OH, M = 46.07g/mol) and Glacial acetic acid (Mw = 60.05 g/mol) was purchased from Chemical Company (Iasi, Romania). The BBB artificial parallel membrane permeability assay kit (PMBBB-096) were purchased from BioAssay (Hayward, CA, USA). The glioma cell line C6 (product code CCL-107) was purchased from CLS. The V79-4 fibroblast cell line (product code CCL-93) was obtained from ATCC (Manassas, VA, USA).

### 4.2. Obtaining Methods

The process for obtaining OG and its characterization is thoroughly detailed in the paper [52].

#### 4.2.1. Preparation of Low-Molecular-Weight Protamine (LMWP)

An enzymatic strategy was employed to modify protamine to prepare LMWP [53]. Protamine and thermolysin, at a 1:100 ratio, were incubated in a 20 mM phosphate buffer solution (PBS) at pH 8, with stirring for 3.5 h at 40 °C in the dark. To inhibit proteolytic activity, 50 mM EDTA was added. The resulting product was purified using a chromatographic column packed with Sephadex G-25, and elution was carried out using 20 mM PBS at pH 8. Protein concentrations in 1 mL samples were measured using the Lowry method, and the purified product was subsequently lyophilized. Twelve 1 mL fractions of modified protamine were collected during the separation process, and a ninhydrin assay was conducted on selected fractions. A schematic representation of the protamine modification process is provided in Figure S1.

#### 4.2.2. Obtaining Nanoparticles with and Without Encapsulated Drugs by the Self-Assembly Method

An improved HSA self-assembly method was used to obtain nanoparticles. HSA is a globular protein with hydrophobic domains inside and hydrophilic domains on its surface. When β-mercaptoethanol is added to the HSA solution, it reduces the disulfide bonds, exposing the hydrophobic domains to interact with hydrophobic drugs; ultimately, the system self-assembles into nanoparticles [124]. Essentially, in the aqueous solution of HSA or HSA–LMWP (60 mg in 5 mL of 0.1 M acetate buffer solution, pH 3.5), mercaptoethanol (at 10 mM) was added, and the solution was stirred for 30 min. Subsequently, varying amounts of OG were dissolved in 10 mL of 0.1 M acetate buffer solution and incorporated into the protein solution while stirring to obtain different molar ratios between the protein’s amine groups and the aldehyde groups of the OG. The solution was stirred slowly at 250 rpm for 24 h to ensure that the crosslinking reaction occurred. After 24 h, the amounts of bioactive principles (TMZ, 10 mg; curcumin, 20 mg; or TMZ/curcumin, 5 mg/10 mg) were dissolved in 20 mL of dimethyl sulfoxide (DMSO) and added to the solution mixture dropwise. The resulting solution was dialyzed for 24 h against bidistilled water to remove mercaptoethanol and DMSO. Consequently, drug-loaded HSA nanoparticles were obtained through self-assembly. Following dialysis, the suspensions containing drug-loaded nanoparticles were lyophilized. Table 1 shows the experimental program.

#### 4.2.3. Determination of Free Amine Groups Using the Ninhydrin Solution Test

The ninhydrin test was employed to measure the number of free amino groups in proteins. A calibration curve was plotted using glutamic acid as a standard (see Figure S4). Subsequently, the free amino groups in HSA, protamine sulfate, and LMWP were quantified. A stock protein solution in 0.1 M acetate buffer at pH 5.6 was prepared with a concentration of 0.1%. From this, albumin solutions were prepared at various concentrations (in µg/mL). The results were expressed as moles of amino groups per gram of protein. To determine the free amino groups, the following protocol using ninhydrin was employed: 1 mL of the protein solution at different concentrations was mixed with 1 mL of 2% ninhydrin solution in a test tube, and the mixture was heated at 95 °C for 30 min. After cooling, 8 mL of an ethanol/distilled water solution (1:1 volume ratio) was added, and the absorbance of the solutions was measured at 570 nm using a UV spectrophotometer. The proteins’ moles of free amino groups were determined referencing the previously established glutamic acid calibration curve. The results are presented as mean values ± standard deviation. The calibration curve for glutamic acid, used as a standard, is shown in Figure S4.

#### 4.2.4. Modification of Human Serum Albumin Using Low-Molecular-Weight Protamine

The thiol group of albumin was activated using the bifunctional compound succimidyl-4-(N-maleimidomethyl)-cyclohexane-1-carboxylate (SMCC). A total of 37 mg of SMCC was dissolved in 10 mL of dimethylsulfoxide (DMSO), resulting in a concentration of 3.7 mg/mL SMCC. The HSA solution was prepared by dissolving 1 g of protein in 50 mL of PBS at a concentration of 20 mM, pH 7.2. The HSA solution was kept in contact with the SMCC solution at room temperature while stirring (300 rpm) for 1 h. Afterward, the solution was purified using a chromatographic column packed with Sephadex G-25. To the purified and activated HSA solution, LMWP (0.1 g, dissolved in 5 mL of PBS at 20 mM, pH 7.2) was added, and the mixture was stirred in the dark for 24 h. Finally, the solution was dialyzed against bidistilled water using 12 kDa cellulose dialysis membranes for 12 h before lyophilization.

### 4.3. Characterization Methods

#### 4.3.1. ^1^H NMR Nuclear Magnetic Resonance Spectroscopy

Nuclear magnetic resonance spectroscopy (Bruker Avance Neo 600, Bruker inc, Billerica, MA ,USA) was used to investigate the structure of HSA and protamine before and after modification. The samples were prepared by dissolving them in deuterated water and then analyzed using the ^1^H NMR technique.

#### 4.3.2. FT-IR Spectroscopy

FT-IR spectra were recorded for protamine, LMWP, HSA, and LMWP-modified HSA and albumin dry nanoparticles obtained through self-assembly with two co-encapsulated drugs. The spectra were collected at the sample interface using a Cary 630 FTIR instrument (Agilent Technologies, Santa Clara, CA, USA) with ATR over a frequency range of 4000 to 400 cm^−1^ and a resolution of 4 cm^−1^.

#### 4.3.3. Determination of Average Particle Diameter

The average diameter of the nanoparticles with immobilized drugs and their size distribution were analyzed using laser beam diffractometry with a SHIMADZU-SALD 7001 diffractometer (Shimadzu Corporation, Kyoto, Japan). Measurements were conducted on nanoparticle suspensions in double-distilled water 24 h after preparation. Before measurements, the suspensions were sonicated in a Bandeli Sonorex sonicator (Bandelin electronic GmbH, Berlin, Germany) bath for 10 min at room temperature. The determinations were made in triplicate, and the results are presented as the average diameter ± standard deviation. In this analysis, we determined the average particle size (D50), along with the D25 and D75 values, which allowed us to calculate an alternative dispersity parameter, namely, SPAN (Equation (1)). We want to clarify that the SALD method does not directly provide values for the polydispersity index (PDI) and zeta potential (ZP). Additionally, we did not conduct any supplementary measurements using dynamic light scattering (DLS) at this stage, so these values are not included in the table. However, to assess the degree of particle dispersity, we calculated an alternative SPAN index using the following formula:

(1) $$SPAN=\frac{\left(D_{75}-D_{25}\right)}{D_{50}}$$

#### 4.3.4. Scanning Electron Microscopy

Scanning electron microscopy (SEM) was employed to analyze the morphology of drug-loaded nanoparticles. A HITACHI SU 1510 (Hitachi High-Tech Corporation, Tokyo, Japan) scanning electron microscope was used to perform this analysis. Before analysis, the dried nanoparticles were metallized with gold, achieved by sputter deposition of a thin layer using a Cressington 108 automatic coater (Cressington Scientific Instruments Ltd., Watford, UK) to enhance conductivity, prior to SEM imaging.

#### 4.3.5. Evaluation of the Encapsulation Efficiency (Ef%) for Temozolomide and Curcumin

Before determining the encapsulation efficiency of drugs in nanoparticles, the samples were accurately weighed, and the bioactive compounds were extracted using ethanol. A suspension of nanoparticles, weighing 10 mg, was dispersed in 3 mL of ethanol. This suspension was introduced into cellulose dialysis membranes with a 12 kDa porosity (Carl Roth GmbH & Co. KG, Karlsruhe, Germany), which were then placed in Erlenmeyer flasks (Adrian Sistem SRL, Bucharest, Romania) containing 40 mL of ethanol. The extraction of the active principle was conducted in the dark at a temperature of 30 °C for 24 h. The amount of active principle was determined spectrophotometrically using a nanodrop spectrophotometer (ND-1000, Thermo Fisher, Waltham, MA, USA), and it was quantified based on the calibration curves of the active principles in ethanol that were previously drawn and shown in Figure S5. The encapsulation efficiency was determined using Equation (2):

(2) $$Ef=\frac{Mexp}{Mi}\times 100$$

Mexp indicates the weight of the drug obtained through extraction in ethanol, measured spectrophotometrically, while Mi refers to the initial weight of the encapsulated drug.

#### 4.3.6. The Study of Release Kinetics for Active Principles Co-Encapsulated in HSA-Based Nanoparticles in Two Different pH Environments Until Equilibrium

The release kinetics of the active compounds from the nanoparticles were evaluated under two different pH conditions: phosphate-buffered solution (PBS) at pH 7.4, which represents the physiological blood pH, and acetate buffer solution (ABS) at pH 4, simulating the acidic environment of brain tumors. Nanoparticles co-encapsulating curcumin and TMZ were dispersed in 5 mL of the corresponding release medium and placed into a 12 kDa dialysis membrane. These samples were then transferred to Erlenmeyer flasks containing 40 mL of 0.1 M PBS at either pH 7.4 or pH 4. The flasks were maintained at 37 °C in a water bath, continuously stirred at 300 rpm, and kept in the dark. At regular intervals, 1 mL aliquots were collected to quantify the released curcumin and TMZ. The concentration of curcumin was determined spectrophotometrically at a wavelength of 425 nm, while TMZ was measured at 327 nm using a Nanodrop spectrophotometer.

Since the drugs are hydrophobic and poorly soluble in aqueous media, 1% (*w*/*w*) Tween 80 was added to the buffer solutions to facilitate the release process.

Tween 80 (1% *w*/*v*) was added to the release medium to ensure the dispersion of hydrophobic compounds and to prevent their precipitation during analysis. The use of nonionic surfactants, such as Tween 80, is common in the release studies of hydrophobic compounds because it helps improve their solubility and prevent precipitation. It is important to note that the presence of a surfactant may increase the apparent solubility, which could influence the release efficiency values obtained. This factor was considered, and the data were interpreted solely within the context of the experimental conditions described in this article. Based on the release curves previously drawn in PBS 0.1 M and acetate buffer solution 0.1 M (both with 1% Tween), the amount of active substance released (curcumin or TMZ) was determined. The release kinetics were studied until equilibrium was reached for both active principles encapsulated in nanoparticles. The calibration curves had the following equations: For curcumin in a mixture with TMZ at pH 7.4: y = 0.0125x, R^2^ = 0.9996 (for curcumin) and y = 0.0055x, R^2^ = 0.9992 (for TMZ); at pH 4: y = 0.0152x, R^2^ = 0.9989 (for curcumin) and y = 0.0077x, R^2^ = 0.9994 (for TMZ).

Additionally, the Ritger–Peppas kinetic model can help clarify the transport and release mechanisms from the polymer matrix [87]:

(3) $$\frac{M_{t}}{M_{\infty }}=k*t^{n}$$

where M_t_ represents the weight of the drug released at time t, M_∞_ signifies the weight of the drug released as time approaches infinity, k is a constant, and n denotes the diffusion exponent.

#### 4.3.7. Evaluation of Cell Apoptosis Induced by Drug-Loaded Nanoparticles

Apoptosis, also known as programmed cell death, is a natural process for eliminating cells. It involves the formation of membrane vesicles, chromatin condensation, and DNA fragmentation. Annexin V, which is a calcium-dependent protein, binds with high affinity to phosphatidylserine (PS), which moves from the inner to the outer membrane during apoptosis. When Annexin V is fluorescently labeled, this PS exposure can be detected using flow cytometry. The presence of PS signals macrophages to clear the cell through phagocytosis. To distinguish viable, apoptotic, and necrotic cells, a combination of Annexin V and propidium iodide is used, which evaluates cell membrane integrity [125]. In late apoptotic and necrotic cells, both the plasma and nuclear membrane integrity is compromised, enabling propidium iodide to penetrate the membrane, bind to nucleic acids, and produce red fluorescence [126]. Cells were cultured in 24-well plates at a density of 50,000 cells per well to create a monolayer. After 24 h of incubation, test compounds (free drugs or nanoparticle formulations) were applied at a concentration of 7.5 µg/mL and incubated for an additional 24 h. Flow cytometry was used to process and analyze the cells according to the manufacturer’s instructions for the assay kit. For interpretation, cells that were negative for both propidium iodide (PI) and Annexin V were classified as viable; cells that were Annexin V-positive and PI-negative were considered apoptotic; and cells that were positive for both markers were identified as necrotic.

#### 4.3.8. Models of the Blood–Brain Barrier and Evaluation of the Permeability of Functionalized Nanoparticles with Co-Encapsulated Drugs

Membrane permeability is a key parameter in evaluating the potential of compounds as drug candidates, as many therapeutics must overcome cell membranes to reach their site of action. Assessing a compound’s ability to permeate biological membranes passively is therefore essential. While cell-based assays are commonly used for this purpose, they are often costly and time-consuming. The parallel artificial membrane permeability assay (PAMPA) offers a rapid and cost-effective alternative for evaluating passive permeability. Specifically, the PMBBB-096 (BioAssay Systems Hayward, CA, USA) kit supports in vitro assessment of BBB permeability. A working method adapted from Muller et al. [127] was modified. Solutions of curcumin and TMZ were prepared in dimethyl sulfoxide (DMSO) at a concentration of 2 mM and then diluted with 0.01 M PBS (pH 7.4) to prepare the drug solution for the donor compartment, resulting in a final concentration of 500 μM in 5% DMSO. Three hundred microliters (300 μL) of 0.01 M PBS with 5% DMSO was added to the acceptor plate (96-well PTFE acceptor plates) (BioAssay Systems Hayward, CA, USA). The donor plate (96-well polycarbonate filter-based donor plates, pore size 0.45 μm) was coated with 5 μL of lyophilized brain lipid extract solution, made by dissolving the brain extract in 400 μL of dodecane. A volume of 200 μL from the 500 μM solution containing the tested active compounds was added to the lipid-coated donor plate. The donor plate was then carefully placed over the acceptor plate, covered with moistened filter paper and a lid to prevent evaporation, and incubated at 37 °C for 18 h. In parallel, 200 μM equilibrium standards were prepared in separate Eppendorf tubes for each test and control sample. In Eppendorf tubes, 80 μL of test solutions or the control standard at a concentration of 500 μM were mixed with 120 μL of PBS solution containing 5% DMSO. The blank was prepared using the PBS solution with 5% DMSO. The next day, equilibrium standards and the blank solution were utilized, and the acceptor compartment’s solution was analyzed. Results were measured using a Nanodrop spectrophotometer at varying wavelengths: 327 nm for TMZ, 425 nm for curcumin, 270 nm for the Low-Permeability Control Standard, and 250 nm for the High-Permeability Control Standard. Based on the absorbance value determined for each respective test sample and the permeability control standard, the permeability rate (Pe) was calculated using the following Equation (4):

(4) $$Pe=C\times -\ln\left(1-\frac{{OD}_{A}}{{OD}_{E}}\right),cm/s$$

where OD_A_ represents the absorbance of the acceptor plate solution minus the absorbance of the blank, and OD_E_ indicates the absorbance of the equilibrium standard minus the absorbance of the blank. Using an 18 h incubation, C = 7.72 × 10^−6^. Solutions of TMZ and unencapsulated curcumin, each with a final concentration of 500 μM in 500 μL, were utilized for this assay. Oxidized gellan crosslinked HSA nanoparticles, obtained through self-assembly, contained curcumin and TMZ. The nanoparticles used for this assay contained 500 μM curcumin and were suspended in 500 μL of PBS solution with 5% DMSO.

#### 4.3.9. Assessment of the Antioxidant Properties of Curcumin Enclosed in Nanoparticles

With some modifications, the method was previously detailed by Mensor et al. [128] and Choi et al. [129]. A stock solution of curcumin (10 mg/50 mL ethanol) was diluted to achieve concentrations ranging from 5 to 30 μg/mL. In the test tubes, 2 mL of each curcumin solution was mixed with 2 mL of DPPH solution (0.1 mM in ethanol). The samples were vortexed for 20 to 30 s, and their absorbance was measured at 517 nm after 40 min using a UV spectrophotometer (BOECO S-22) (BOECO, Hamburg, Germany). Ethanol is used for calibration, and ascorbic acid serves as a standard for antioxidant activity. The percentage of DPPH inhibition (I%) was calculated with Equation (5):

I% = 100 − [(A_s_ − A_b_) × 100/A_c_] (5)

where A_s_ represents the absorbance of the sample, consisting of 2 mL of curcumin solution at varying concentrations combined with 2 mL of DPPH solution; A_b_ refers to the blank, made up of 2 mL of ethanol mixed with 2 mL of curcumin solution at different concentrations; and A_c_ denotes the control, which consists of 2 mL of ethanol combined with 2 mL of DPPH solution.

In order to evaluate the antioxidant activity of curcumin incorporated into HSA-based nanoparticles crosslinked with OG, both UV-irradiated (at a wavelength of 365 nm for 30 min) and non-irradiated samples were used. Nanoparticles containing curcumin or curcumin/TMZ, at specified curcumin concentrations, were dispersed in 25 mL of ethanol and then successively diluted to achieve the desired concentrations (5–30 μg/mL). The percentage of DPPH inhibition for each sample type was determined, and the IC_50_ values were calculated based on the concentration (μg/mL) vs. inhibition plots. Ascorbic acid was used as a standard. All measurements were performed in triplicate at room temperature.

#### 4.3.10. Evaluation of Cytotoxicity of Drug-Encapsulated in Nanoparticles Using the MTT Assay

In vitro studies were conducted on the normal cell line V79-4 (ATCC CCL-93), which consists of Chinese hamster lung fibroblasts (Cricetulus griseus), and the tumor line C6 (ATCC CCL-107), derived from glial cells of a Rattus norvegicus glioma. V79-4 cells were cultured in Dulbecco’s Modified Growth Medium (DMEM) from PAN-Biotech GmbH, Aidenbach, Germany, while C6 cells were maintained in RPMI-1640 medium (ATCC 30-2001). In both cases, the growth media were supplemented with 10% fetal bovine serum (SFB, Euroclone S.p.A., Milan, Italy) and 1% antibiotic solution, containing penicillin at 100 μg/mL and streptomycin at 100 IU/mL (Capricorn Scientific GmbH, Ebsdorfergrund, Germany), maintained at a temperature of 37 °C in a humidified environment with 5% CO_2_ [130]. Products tested: Albumin nanoparticles obtained through self-assembly and crosslinking with OG were evaluated. The concentrations of the doses used were 1.875, 3.75, 7.5, and 15 µg/mL (µg nanoparticles/mL). For each set of in vitro determinations, both curcumin and TMZ, along with 0.1% DMSO utilized for the solubilization of curcumin and TMZ, were analyzed. The samples used to assess the cytotoxicity of HSA nanoparticles crosslinked with the two encapsulated drugs included nanoparticles (1:0.75-molar ratio) of MTG2, MTCG2, NTCG2, and MG2, as well as the free, unencapsulated drugs curcumin and TMZ.

- Determination of cell viability—MTT method

The 3-(4,5-dimethylthiazol-2-yl)-2,5-diphenyltetrazolium bromide (MTT) colorimetric method was utilized, as modified from Laville et al. (2004) [131], Mosmann (1983) [132], and van Meerloo et al. (2011) [133]. This method relies on the conversion of MTT, a water-soluble yellow compound, into dark blue, water-insoluble formazan by the mitochondrial dehydrogenases of living cells. Cells were cultured in 96-well plates and treated for 48 h. After treatment, the medium was removed, the cells were washed with phosphate-buffered saline (PBS), and then incubated with 100 μL of fresh medium and 10 μL of MTT (5 mg/mL) for 3 h at 37 °C. The resulting formazan crystals were solubilized with 50 μL of DMSO, and absorbance was measured at 570 nm using the EZ Read 400 reader (Biochrom, Cambridge, UK). The intensity of the resulting color directly correlates with the number of viable cells, and viability was calculated using Equation (6):

Cell viability (%) = E_sample_/E_control_ × 100, where E = extinction (6)

#### 4.3.11. Analysis of the Hemocompatibility of Nanoparticles

In order to evaluate the hemocompatibility of the synthesized nanoparticles, we used the spectrophotometric method [134]. Blood (5 mL) was centrifuged at 2000 rpm for 5 min (centrifuge model MIKRO 220 Classic (Unrefrigerated), Andreas Hettich GmbH-Hettich Group, Tuttlingen, Germany); the plasma was discarded, and the sedimented red blood cells (RBCs) were washed with saline solution and then diluted in 25 mL of saline solution. For the experiment, 0.5 mL of the RBC suspension was mixed with 0.5 mL of the nanoparticle suspension in saline, resulting in final concentrations of 25, 50, and 100 μg/mL. Controls included 2% Triton X-100 (positive control, 100% lysis) (Sigma-Aldrich, Darmstadt, Germany) and 0.9% NaCl saline solution (negative control, 0% lysis) (Sigma-Aldrich, Darmstadt, Germany), both at a volume equivalent to the RBC suspension. The samples were incubated at 37 °C for 90 and 180 min, with shaking every 30 min to maintain the resuspension of the RBCs and nanoparticles. Following incubation, the samples were centrifuged at 2000 rpm for 5 min, and the absorbance of oxyhemoglobin in the supernatant was measured spectrophotometrically at 540 nm. The degree of hemolysis was calculated as shown in Equation (7):

(7) $$Hemolysis=\frac{\left({Abs}_{sample}-{Abs}_{negative\;control}\right)}{\left({Abs}_{positive\;control}-{Abs}_{negative\;control}\right)}\times 100$$

#### 4.3.12. Analysis of pH Stability in Drugs Encapsulated Within Nanoparticles

The method for determining the stability of free curcumin at various pH levels was adapted from [135]. First, 10 mg of curcumin was dissolved in 100 mL of ethanol, and 1 mL of this stock solution was utilized to prepare a 25 mL solution with a curcumin concentration of 4 μg/mL at pH values of 3, 6.8, 7.4, and 9. The samples were maintained at 37 °C in the dark, in closed containers, for 8 h. The curcumin concentration in these solutions was determined spectrophotometrically using a calibration curve at 425 nm, every hour for 8 h. To assess the pH stability of encapsulated curcumin, samples with modified HSA and those without modified HSA were selected. A total of 2 mg of dry HSA-based nanoparticles, modified or not with LMWP and loaded with curcumin and TMZ, were added to 2 mL of solution at different pH levels. The particles were maintained at 37 °C in an oven (AG-System, F.lli Galli G. & P., Milano, Italia) protected from light and air exposure. Samples of 100 μL were taken every hour for up to 8 h, and 900 μL of ethanol was added to extract curcumin from the nanoparticles. The amount of curcumin found in the samples was then determined using a Nanodrop spectrophotometer. The curcumin concentration extracted from the samples was determined spectrophotometrically using the calibration curve of curcumin in ethanol at a wavelength of 425 nm. From these results, a straight line was drawn between the logarithm of the concentration of undegraded curcumin and time, allowing for the determination of both the degradation rate constant k and the drug’s half-life at degradation t_1/2_ (Equation (8)).

(8) $$t_{1/2}=\frac{ln2}{k}\sim \frac{0.693}{k}$$

The half-life (t_1/2_) for first-order reactions depends solely on the degradation rate constant, k, and is independent of the substance’s initial concentration. All measurements were conducted in triplicate, and the results are reported as average values ± standard deviation (STDev).

#### 4.3.13. Study of Light Stability in Drugs Encapsulated in Nanoparticles

The light stability of free and encapsulated curcumin was determined spectrophotometrically using a Nanodrop spectrophotometer. The analyzed HSA nanoparticles were weighed and then exposed to air and natural light for 30 days in Petri dishes. At various time points, nanoparticles from each sample were collected and weighed, and the encapsulated curcumin was extracted using ethanol (1.5 mL). The absorbance was measured at 425 nm, and based on the calibration curve, the amount of curcumin in the particles was calculated. The results were expressed as milligrams of curcumin per gram of particles. All determinations were performed in triplicate, and the results are presented as mean values ± standard deviation (STDev).

#### 4.3.14. Thermogravimetric Analyses

Thermogravimetric analyses measured the weight loss of the samples as a function of temperature. These measurements were performed with a TA Instruments Q600 (Waters, New Castle, DE, USA) analyzer at atmospheric pressure (100 mL/min) with a heating rate of 10 °C/min from ambient temperature to 700 °C. The samples had a mass between 6 and 9 mg, and to obtain comparable data, the operating parameters were kept constant throughout the experiments.

#### 4.3.15. Statistical Analyses

Results are presented as mean ± standard deviation. Student’s *t*-test was used to assess unequal variance. A two-tailed *p*-value less than 0.05 was considered significant.

## Figures and Tables

**Table 1:** Total moles of free amine groups in standard protamine versus enzymatically modified low-molecular-weight protamine.

Sample	Number of Moles of NH_2_ Groups × 10^−4^/g Protein
Protamine	5.27 ± 1.22
Fraction 4	8.31 ± 1.58
Fraction 5	11.19 ± 3.47
Fraction 6	13.91 ± 3.47
Fraction 7	16.3 ± 2.46
Fraction 8	18.75 ± 0.46
Fraction 10	19.3 ± 0.56
Fraction 11	23.57 ± 1.5

**Table 2:** Experimental program.

Sample Code *	CHO/NH_2_ Ratio	HSA (mg)	OG (mg)	Drug(s) Encapsulated	Crosslinked with OG	Functionalized with LMWP
NT0	0	60	0	TMZ	No	No
NCT0	0		0	TMZ/curcumin	No	No
NC0	0		0	curcumin	No	No
NG1	0.5/1		10	No	Yes	No
NG2	0.75/1		15	No	Yes	No
NG3	1/1		20	No	Yes	No
NTG1	0.5/1		10	TMZ	Yes	No
NTG2	0.75/1		15	TMZ	Yes	No
NTG3	01-Jan		20	TMZ	Yes	No
NTCG1	0.5/1		10	TMZ/curcumin	Yes	No
NTCG2	0.75/1		15	TMZ/curcumin	Yes	No
NTCG3	1/1		20	TMZ/curcumin	Yes	No
NCG1	0.5/1		10	curcumin	Yes	No
NCG2	0.75/1		15	curcumin	Yes	No
NCG3	1/1		20	curcumin	Yes	No
MT0	0		0	TMZ	No	Yes
MCT0	0		0	TMZ/curcumin	No	Yes
MC0	0		0	curcumin	No	Yes
MG2	0.75/1		15	No	Yes	Yes
MTG2	0.75/1		15	TMZ	Yes	Yes
MTCG1	0.5/1		10	TMZ/curcumin	Yes	Yes
MTCG2	0.75/1		15	TMZ/curcumin	Yes	Yes
MTCG3	01-Jan		20	TMZ/curcumin	Yes	Yes
MCG2	0.5/1		10	curcumin	Yes	Yes

* The number of moles of amine groups in albumin was found to be 3.79 × 10^−5^ in a sample of 60 mg. The number of moles of aldehyde groups in oxidized gellan varied depending on the molar ratio of CHO to NH2. The values were as follows: at a molar ratio of 0.5:1, the number of moles of aldehyde groups was 1.89 × 10^−5^; at a molar ratio of 0.75:1, it was 2.84 × 10^−5^; and at a molar ratio of 1:1, the number was 3.79 × 10^−5^.

**Table 3:** The average particle size and dispersion index (SPAN) of the samples determined using SALD analysis.

Sample	Average Diameter (nm)	SPAN
NT0	118 ± 1.5	0.44 ± 0.01
NTC0	124.7 ± 2.9	0.35 ± 0.014
NC0	83.7 ± 3.8	0.39 ± 0.02
NTG1	119 ± 0.81	0.42 ± 0.009
NTCG1	112 ± 1.0	0.41 ± 0.035
NCG1	91.5 ± 0.5	0.45 ± 0.032
NTG2	134.5 ± 11.3	0.36 ± 0.026
NTCG2	143.3 ± 3.9	0.56 ± 0.07
NCG2	122.7 ± 0.9	0.35 ± 0.003
NTG3	145.3 ± 0.5	0.39 ± 0.008
NTCG3	189.7 ± 8.3	0.38 ± 0.006
NCG3	292.3 ± 0.5	0.26 ± 0.0004
MT0	78	0.45
MCT0	81 ± 1.2	0.31 ± 0.02
MC0	96.7 ± 0.5	0.36 ± 0.003
MTG2	67	0.42 ± 0.007
MTCG2	79 ± 5.5	0.43 ± 0.03
MCG2	104	0.375

**Table 4:** Immobilization efficiency (Ef%) for particles obtained through self-assembly encapsulated with temozolomide (TMZ) and curcumin (CURC).

Sample	EF% TMZ	EF% CURC
NT0	18.6 ± 2.06	0
NTG1	18.64 ± 1.1	0
NTG2	17.44 ± 1.94	0
NTG3	15.86 ± 0.93	0
NTC0	28.07 ± 3.22	73.04 ± 1.24
NTCG1	33.69 ± 0.78	94.68 ± 0.71
NTCG2	24.29 ± 2.35	66.6 ± 5.48
NTCG3	15.88 ± 0	48.71 ± 0.36
NC0	0	92.35 ± 1.86
NCG1	0	91.05 ± 0.45
NCG3	0	34.11 ± 0.41
MT0	14.74 ± 0.7	0
MTG2	23.61 ± 0	0
MC0	0	92.86 ± 0.4
MCG2	0	88.92 ± 0
MTC0	33.92 ± 3.01	69.73 ± 3.02
MTCG2	31.29 ± 0.7	82.51 ± 7.8

**Table 5:** The values of release efficiency and the exponential factor n are determined from studying drug release kinetics.

Sample	Release Efficiency, Ef% Curcumin	Release Efficiency, Ef% TMZ	Exponential Factor, n (Curcumin)	R^2^ (Curcumin)	Exponential Factor, n (TMZ)	R2 (TMZ)
NTCG1-4	22.76 ± 0.5	68.36 ± 1.9	0.25	0.8459	0.35	0.983
NTCG2-4	20.24 ± 0.43	65.86 ± 2.6	0.26	0.8554	0.38	0.9505
NTCG3-4	13.16 ± 0.5	57.71 ± 0.8	0.3	0.967	0.402	0.9781
NTCG1-7.4	58.66 ± 3.46	63 ± 1.7	0.61	0.983	0.4135	0.973
NTCG2-7.4	56.96 ± 0.5	61.76 ± 0.5	0.6	0.992	0.4746	0.9847
NTCG3-7.4	36.5 ± 0.9	49.08 ± 1.4	0.76	0.9205	0.2837	0.9657
MTCG1-4	59 ± 0.1	80.8 ± 0.6	1.	0.851	0.8749	0.9324
MTCG2-4	38.67 ± 0.5	66.7 ± 3.5	0.42	0.9647	0.7439	0.9282
MTCG3-4	24.44 ± 0.5	61.99 ± 1.95	0.59	0.9774	0.6234	0.9876
MTCG1-7.4	74.77 ± 0.4	72.23 ± 1.3	0.34	0.9784	0.253	0.9751
MTCG2-7.4	60.38 ± 0.4	58.08 ± 0.3	0.31	0.9872	0.2597	0.9795
MTCG3-7.4	63.45 ± 1.1	49.84 ± 0.9	0.34	0.9819	0.2819	0.9733

**Table 6:** IC_50_ (μg/mL) value for drugs such as free curcumin (CURC), free temozolomide (TMZ), and the ones encapsulated in MTCG2, MTG2, and NTCG2 albumin nanoparticles.

Sample	IC_50_ μg/mL for TMZ on the C6 Cell Line	IC_50_ μg/mL for TMZ on the V79-4 Cell Line	IC_50_ for μg/mL for CURC on the C6 Cell Line	IC _50_ μg/mL for CURC on the V79-4 Cell Line	IC_50_ μg/mL for TMZ/CURC on the C6 Cell Line	IC_50_ μg/mL for TMZ/CURC on the V79-4 Cell Line
MTCG2	1.13	2.24	1.8	3.58	2.93	5.81
MTG2	0.51	0.53	0	0	0.51	0.53
NTCG2	1.41	1.85	2.21	2.9	3.62	4.75
TMZ	8.75	16.84	0	0	8.75	16.84
Curcumin	0	0	6.31	10.36	6.31	10.36

**Table 7:** Percentage of undegraded curcumin (%), degradation rate constant (k), and degradation half-life (t1/2) for free curcumin and curcumin encapsulated in crosslinked HSA-based nanoparticles.

Sample	pH	Variation of Undegraded Curcumin (%) over Time				k	t_1/2_ (h)
		0 h	1 h	5 h	8 h		
Free Curcumin	3	100	76	55.7	47.6	0.091	7.7
	6.8	100	76.4	56.8	43.7	0.107	6.5
	7.4	100	77	52.7	41.1	0.119	5.8
	9	100	61	21	8.6	0.301	2.3
MTCG2	3	100	95.8	65.2	53.8	0.0357	19.3
	6.8	100	98.2	83.1	66.9	0.0213	32.5
	7.4	100	99.5	83	82.4	0.013	53.3
	9	100	93.87	64.8	58	0.0322	21.5
NTCG2	3	100	96.8	61.3	55.2	0.0363	19.1
	6.8	100	91.8	77.4	66.6	0.0231	30
	7.4	100	97.5	81.2	75.9	0.0162	42.8
	9	100	99.5	72.8	59.7	0.0331	21.3

**Table 8:** Percentage of curcumin degradation (%) under natural light for free curcumin and curcumin encapsulated in two types of nanoparticles. All samples were exposed to natural light and air for 30 days.

Sample	Percentage of Degradation for Free Versus Encapsulated Curcumin over Time, %				
	Day 3	Day 10	Day 18	Day 26	Day 30
Curcumin	6.8	13.5	16.9	26	32.3
NC0	0.5	11.3	16.9	18.6	18.7
MC0	3	7.3	8.7	10.4	10.7
MTC0	6.2	9.9	12.5	19.7	19.8
NTC0	5.4	3.2	9	12.4	17.5
MTCG2	1	2.7	8.8	9.8	12.8
NTCG2	0.8	7.2	12.3	15.3	16.3
MTCG3	1.9	2.5	5.4	12.6	12.7

## Data Availability

The data presented in this study are available within the article.

## Supplementary Materials

The following supporting information can be downloaded at: https://www.mdpi.com/article/10.3390/gels11090708/s1, Figure S1: Schematic presentation of the method for obtaining low-molecular-weight protamine; Figure S2: UV-Vis absorption spectra for HSA, HSA-SMCC (after purification), LMWP, and HSA–LMWP; Figure S3: Dimensional polydispersity curves for particles created from unmodified HSA with LMWP (obtained through self-assembly that encapsulated curcumin (a), temozolomide (b), and curcumin/temozolomide mixture (c)); Figure S4: Calibration curve of glutamic acid (number of moles of NH2 groups vs. Absorbance); Figure S5: Calibration curves in ethanol for temozolomide at λ = 327 nm (a), for the mixture of curcumin (λ = 425 nm) and temozolomide (λ = 327 nm) (b), and for curcumin (λ = 425 nm); Table S1: Characteristic dimensions and polydispersity index (SPAN) of particles determined based on SALD analysis; Table S2: Effects of various types of HSA-based nanoparticles produced through self-assembly and crosslinking with OG, with or without co-encapsulated active substances, on C6 tumor cells and normal V79-4 cells; Table S3: Drug concentration in each type of nanoparticle used for C6 and V79-4 treatment.
