# Supplementary material for: Development of an Innovative Nanosystem Based on Functionalized Albumin and Oxidized Gellan for the Synergistic Delivery of Curcumin and Temozolomide in the Treatment of Brain Cancer
Source: Gels. 2025 Sep 3;11(9):708. doi: 10.3390/gels11090708 (PMC12469971; doi:10.3390/gels11090708)
Supplement: Supplementary file 1 [file gels-11-00708-s001.zip › Supplementary materials.pdf]

Supplementary materials

The article's title: Development of an Innovative Nanosystem Based on Functionalized Albumin and Oxidized Gellan for the Synergistic Delivery of Curcumin and Temozolomide in the Treatment of Brain Cancer

Authors: Camelia Elena Iurciuc (Tincu), Gabriela Vochița, Daniela Gherghel, Mihai Cosmin-Teodor, Silvia Vasiliu, Ștefania Racoviță, Anca Niculina Cadinoiu, Corina-Lenuța Logigan, Mihaela Hamcerencu, Florin Mitu, Marcel Popa, Ochiuz Lăcrămioara

Figure S1

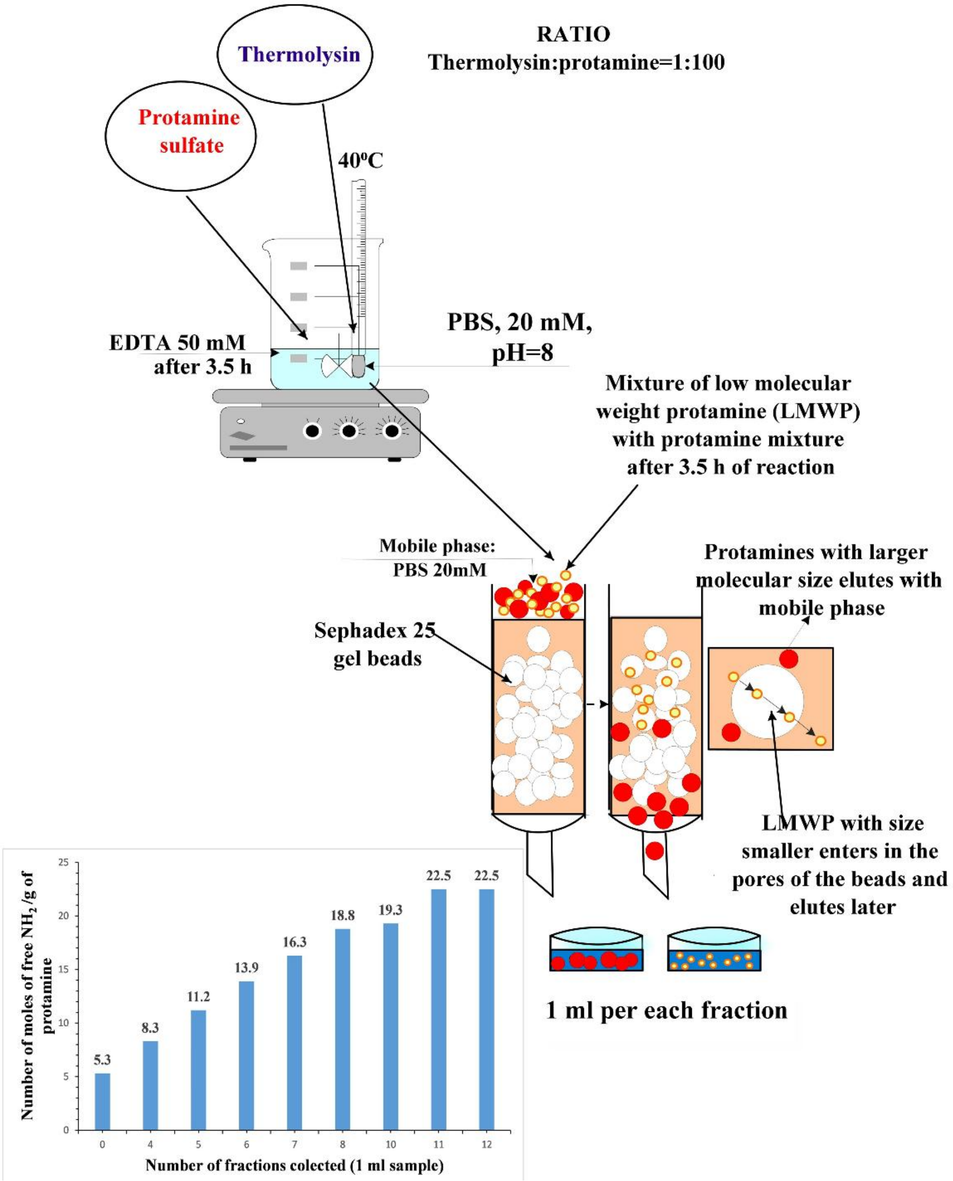

Figure S1. Schematic presentation of the method for obtaining low molecular weight protamine

Figure S2

HSA

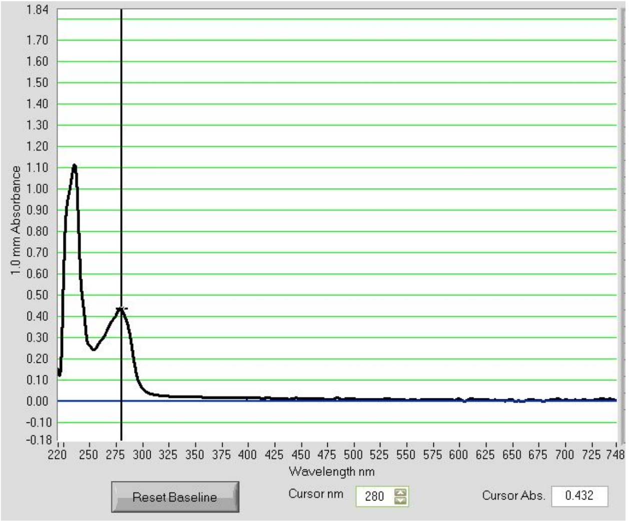

HSA-SMCC

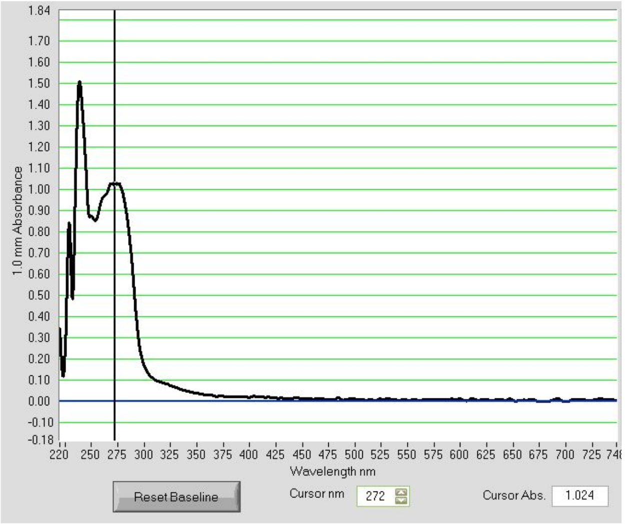

LMWP

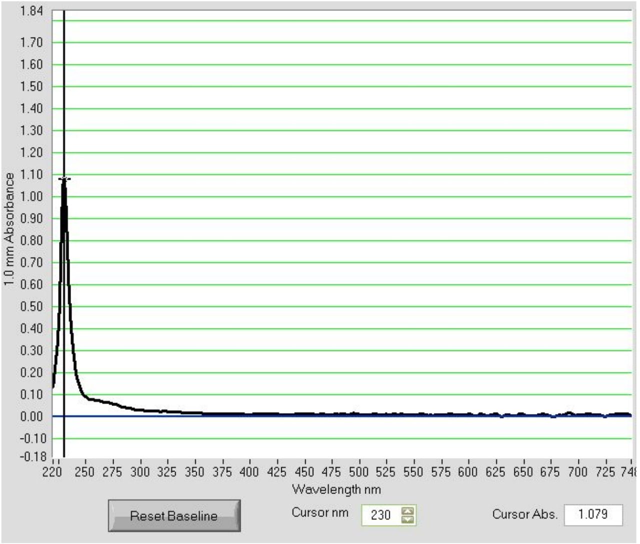

HSA-LMWP

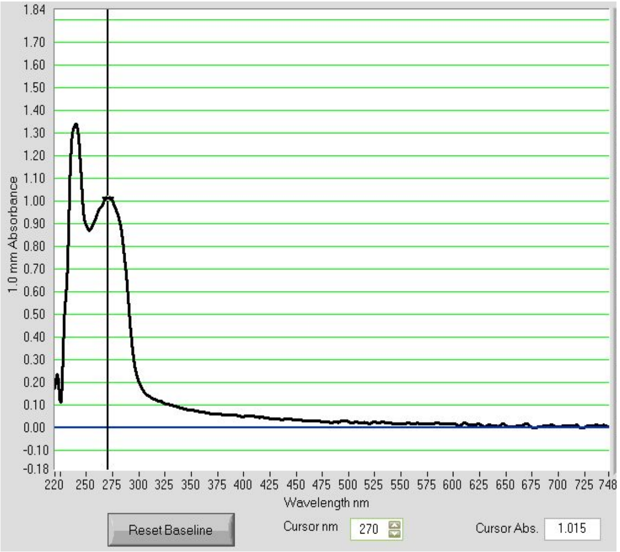

Figure S2. UV-Vis absorption spectra for HSA, HSA-SMCC (after purification), LMWP, and HSA-LMWP.

Figure S3

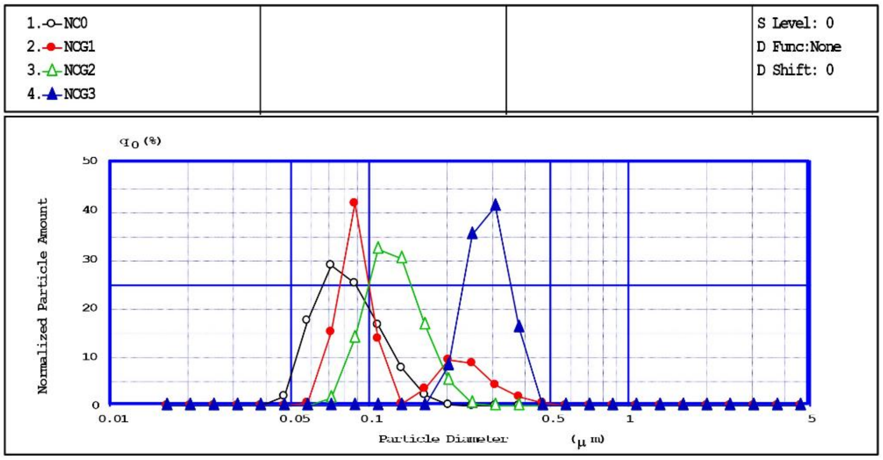

(a)

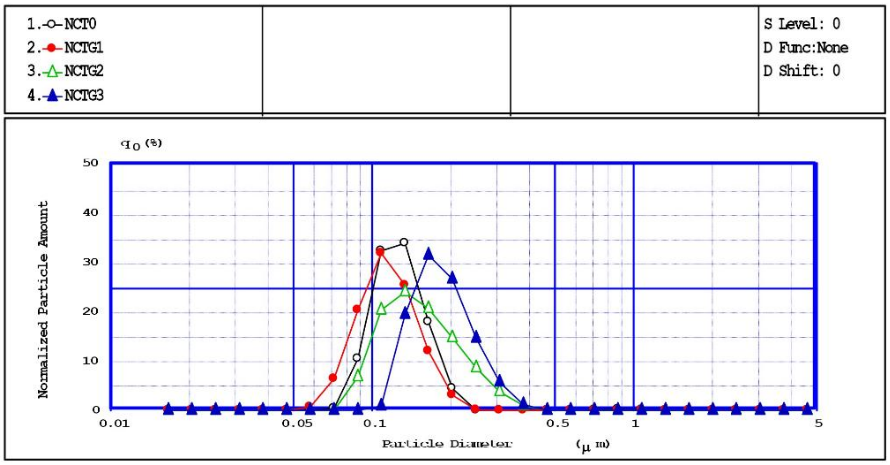

(b)

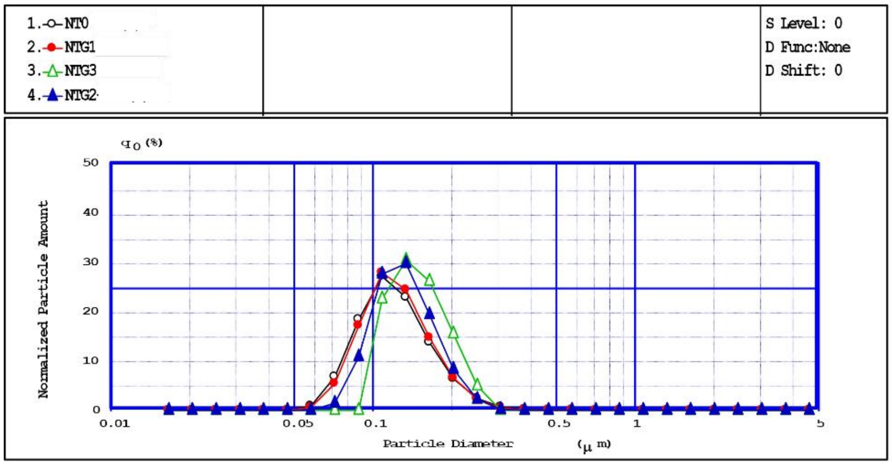

(c)

Figure S3. Dimensional polydispersity curves for particles created from unmodified HSA with LMWP (obtained through self-assembly that encapsulated curcumin (a), temozolomide (b), and curcumin/temozolomide mixture (c)).

Figure S4

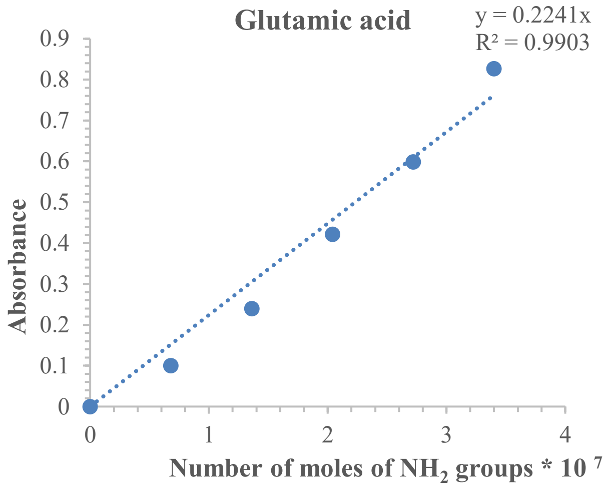

Figure S4. Calibration curve of glutamic acid (number of moles of NH_2 groups vs. Absorbance)

Figure S5

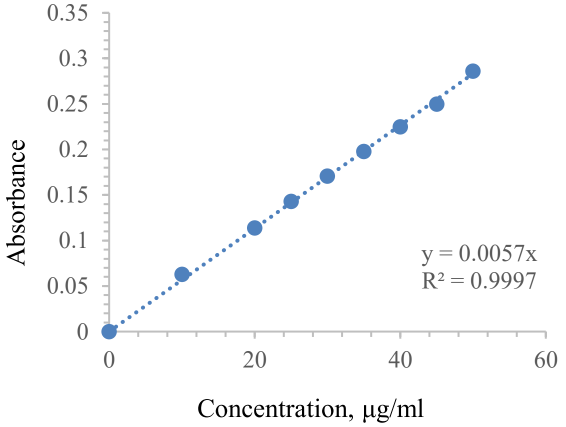

(a)

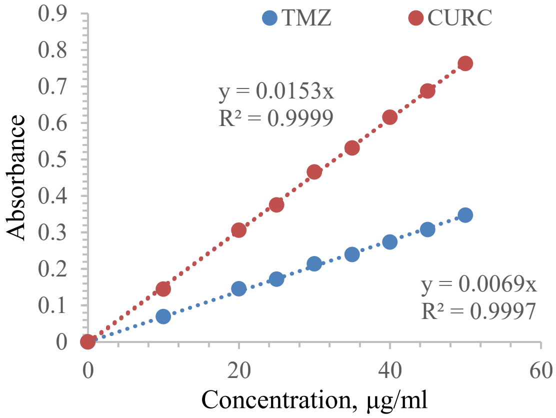

(b)

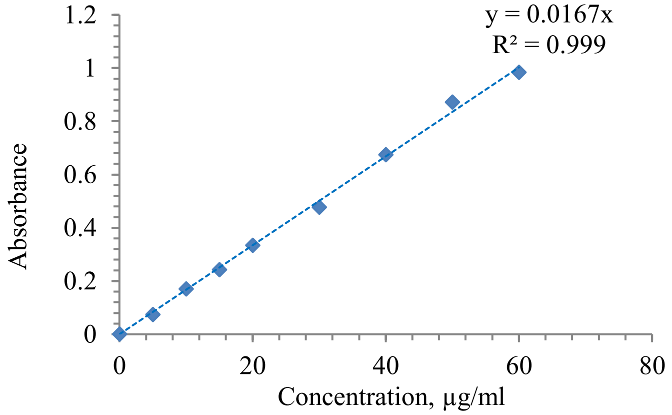

(c)

Figure S5. Calibration curves in ethanol for temozolomide at λ=327 nm (a), for the mixture of curcumin (λ=425 nm) and temozolomide (λ=327 nm) (b), and curcumin (λ=425 nm)

Table S1

Table S1. Characteristic dimensions and polydispersity index (SPAN) of particles determined based on SALD analysis

**Sample**	**D75 (nm)**	**D50 (nm)**	**D25 (nm)**	**SPAN**
NT0	148.5±1.12	118±1.5	96.3±1.5	0.44±0.01
NTC0	149±2.16	124.7±2.9	106±2.2	0.35±0.014
NC0	102±3.56	83.7±3.8	69.7±3.8	0.39±0.02
NTG1	148.7±0.5	119±0.81	98.7±0.9	0.42±0.009
NTCG1	138±1	112±1.0	92.5±2.5	0.41±0.035
NCG1	154.5±33.6	91.5±0.5	82.25±1.9	0.45±0.032
NTG2	161.5±11.3	134.5±11.3	113.8±11.1	0.36±0.026
NTCG2	192.7±9.5	143.3±3.9	111.7±3.9	0.56±0.07
NCG2	147.7±0.9	122.7±0.9	105±0.8	0.35±0.003
NTG3	178.3±0.5	145.3±0.5	121.3±0.9	0.39±0.008
NTCG3	232.7±8.3	189.7±8.3	161±6.5	0.38±0.006
NCG3	336.3±0.5	292.3±0.5	260.3±0.5	0.26±0.0004
MT0	99	78	64	0.45
MCT0	95.3±1.25	81±1.2	70.3±1.7	0.31±0.02
MC0	116.7±0.5	96.7±0.5	82	0.36±0.003
MTG2	83.3±0.5	67	55	0.42±0.007
MTCG2	98.5±5.5	79±5.5	64.5±5.5	0.43±0.03
MCG2	125	104	86	0.375

Table S2

Table S2. Effects of various types of HSA-based nanoparticles produced through self-assembly and cross-linking with OG, with or without co-encapsulated active substances, on C6 tumor cells and normal V79-4 cells.

**C6**	FITC-H- PI-H-	FITC-H- PI-H+	FITC-H+ PI-H-	FITC-H+ PI-H+
	Living cells (%)	Dead cells (%)	Preapoptotic cells (%)	Apoptotic cells (%)
BLANK	94.4	2.49	0.22	2.89
CURC	37.92	14.33	12.11	35.64
TMZ	40.08	16.45	9.88	33.59
MTG2	71.95	2.91	8.13	17.01
MTCG2	72.92	3.06	6.82	17.2
NTCG2	78.15	3.18	6.01	12.66
MG2	84.94	9.77	1.82	3.47

V79-4	FITC-H- PE-H- Living cells (%)	FITC-H- PE-H+ Dead cells (%)	FITC-H+ PE-H- Preapoptotic cells (%)	FITC-H+ PE-H+ Apoptotic cells (%)
BLANK	98.4	0.49	0.14	0.97
CURC	43.47	17.93	11.51	27.09
TMZ	49.36	15.17	10.61	24.86
MTG2	73.11	6.16	9.46	11.27
MTCG2	76.49	1.75	8.08	13.68
NTCG2	80.35	3.77	8.73	7.15
MG2	85.26	9.74	2.8	2.2

Table S3

Table S3. Drug concentration in each type of nanoparticle used for C6 and V79-4 treatment

Sample	Nanoparticle concentrations, µg/ml	Total drug concentration in nanoparticles, µg/ml	CURC concentration, µg/ml	TMZ concentration, µg/ml	Cell viability on C6 cell line, %	Cell viability on V79-4 cell line, %	Percentage of dead cells per C6 cell line, %	Percentage of dead cells per V79-4 cell line, %
MCTG2	1.875	0.195	0.12	0.075	96.36	98.84	3.64	1.16
	3.75	0.39	0.24	0.15	89.65	91.2	10.35	8.8
	7.5	0.78	0.48	0.3	80.77	87.71	19.23	12.29
	15	1.56	0.96	0.6	73.44	85.74	26.56	14.26
MTG2	1.875	0.037	0	0.037	86.97	96.12	13.03	3.88
	3.75	0.074	0	0.074	78.31	90.07	21.69	9.93
	7.5	0.148	0	0.148	69.37	83.25	30.63	16.75
	15	0.296	0	0.296	67.15	71.58	32.85	28.42
Curcumin	1.875	1.875	1.875	0	62.34	75.79	37.66	24.21
	3.75	3.75	3.75	0	55.78	60.86	44.22	39.14
	7.5	7.5	7.5	0	39.16	52.67	60.84	47.33
	15	15	15	0	37.37	41.59	62.63	58.41
TMZ	1.875	1.875	0	1.875	62.3	75.84	37.7	24.16
	3.75	3.75	0	3.75	57.5	66.95	42.5	33.05
	7.5	7.5	0	7.5	52.95	60.36	47.05	39.64
	15	15	0	15	39.05	54.47	60.95	45.53
NTCG2	1.875	0.262	0.16	0.102	90.69	94.68	9.31	5.32
	3.75	0.524	0.32	0.204	81.69	86.04	18.31	13.96
	7.5	1.048	0.64	0.408	78.17	81.27	21.83	18.73
	15	2.096	1.28	0.816	67.86	75.7	32.14	24.3
